# Folic acid functionalization for targeting self-assembled paclitaxel-based nanoparticles†

**DOI:** 10.1039/d2ra06306a

**Published:** 2022-12-12

**Authors:** Eleonora Colombo, Davide Andrea Coppini, Simone Maculan, Pierfausto Seneci, Benedetta Santini, Filippo Testa, Lucia Salvioni, Giovanni Maria Vanacore, Miriam Colombo, Daniele Passarella

**Affiliations:** Dipartimento di Chimica, Università degli Studi di Milano Via Golgi 19 20133 Milano Italy daniele.passarella@unimi.it; Dipartimento di Biotecnologie e Bioscienze, Università degli Studi di Milano Bicocca Piazza della Scienza 2 20126 Milano Italy; Dipartimento di Scienza dei Materiali, Università degli Studi di Milano Bicocca Via Roberto Cozzi 55 20125 Milano Italy

## Abstract

Hetero-nanoparticles self-assembled from a conjugate bearing folic acid as the targeting agent, and another bearing paclitaxel as the active agent are reported. Hetero-nanoparticles containing varying percentages of folic acid conjugates are characterised, and their biological activity is determined.

## Abstract 2

Self-assembled hetero-nanoparticles are obtained by mixing paclitaxel and folic acid conjugates.

## Graphical abstract

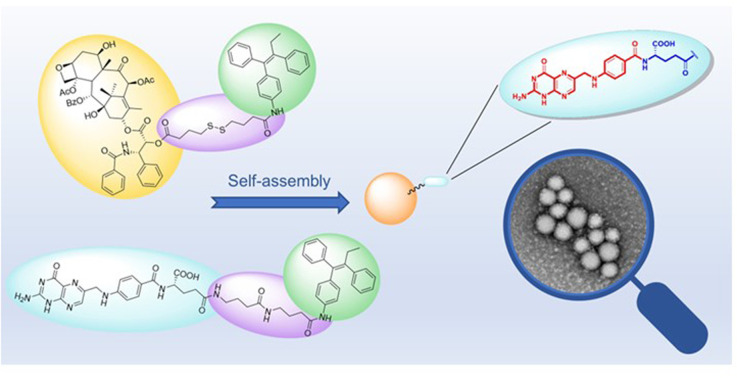

## Introduction

The development of targeted drug delivery nanosystems is a challenging problem, aiming to provide an efficient transport of bioactive molecules and their site-specific release in the microenvironment of diseased tissues. Since several years we have been interested in modifying anticancer and neuroprotective drugs to obtain self-assembling nanoparticles (NPs) that could improve their therapeutic efficiency.

Although traditional, carrier-based NPs have shown excellent progresses and promises in the field of cancer therapy, further improvements are still needed. For example, the drug-loading capacity of such carrier-based NPs is generally low (typically <10 wt%), which greatly reduces an efficient drug accumulation inside tumors and therapeutic efficacy of the released drugs.^1^ Additionally, meanwhile most reported nanocarriers are pharmaceutically inert, because of the sophisticated preparation procedures and excessive chemical treatments, application of these carriers raises concerns regarding their metabolisms, biodegradation, and potential long-term toxicities as well as serious inflammation.^2^ Because of that, self-assembling nanoparticles, which carry the therapeutic molecules by themselves instead of using other inert carriers, are a highly desirable alternative strategy for the development of NPs. They present in fact: (1) high drug-loading capacity; (2) precise control of drug loading because the nanostructures formed from customized individual molecular conjugates; (3) facile adjustment of the physicochemical features of the NPs by simply optimizing the molecular design; (4) avoiding tedious synthetic procedures for obtaining additional carriers and thus no carrier-induced potential cytotoxicity and immunogenicity; (5) enhancement of drug accumulation in tumors.^3^ With such advantages, we believe the development of this kind of nanoparticles will be the main trend in development of nanomedicine for drug delivery. During the years, we reported the synthesis of different lipid–drug conjugates, obtained by a covalent coupling of the drug to biocompatible lipid moieties through a linker. This kind of conjugates is capable to spontaneously assemble in water, forming NPs able to release a payload drug in cellular media.^4–8^ Additional modifications can be made on this simple design in order to obtain hetero-NPs bearing two different drugs (combining different conjugates that present the same lipidic self-assembling inducer);^9,10^ single and dual drug fluorescent hetero-NPs (where in one of the conjugates the drug is substituted by a fluorescent moiety)^11,12^ and NPs formed by self-assembling conjugate dual drugs (in which also the self-assembly inducer is pharmaceutically active).^13,14^ Recently, we focused on further improving these NPs, by exploiting targeted drug delivery through folate-containing hetero-NPs.

A wide variety of receptors, such as transferrin,^15^ death receptor (DR) complexes,^16^ epidermal growth factor receptor (EGFR),^17^ as well as tumor-specific antigens and folate ligands^18^ have been exploited to selectively target tumor tissues with nanodrugs. We focused our attention onto folate receptor α (FRα), which plays an important role in cancer biology. Firstly, this isoform has a limited physiological role in non-malignant tissues after embryogenesis, while it is overexpressed in a variety of cancer types.^19–22^ Moreover, FRα shows high affinity for non-physiological substrates, such as folic acid, compared with other folate transporters, enabling the development of folic acid nanoconjugates. In particular, folic acid has been intensively used for clinic applications,^23–25^ and even though no folate-based NPs have entered the clinic for cancer therapy yet, examples of folate-conjugated nanovehicles have been published in literature in the last years.^26–28^

**Fig. 1:**
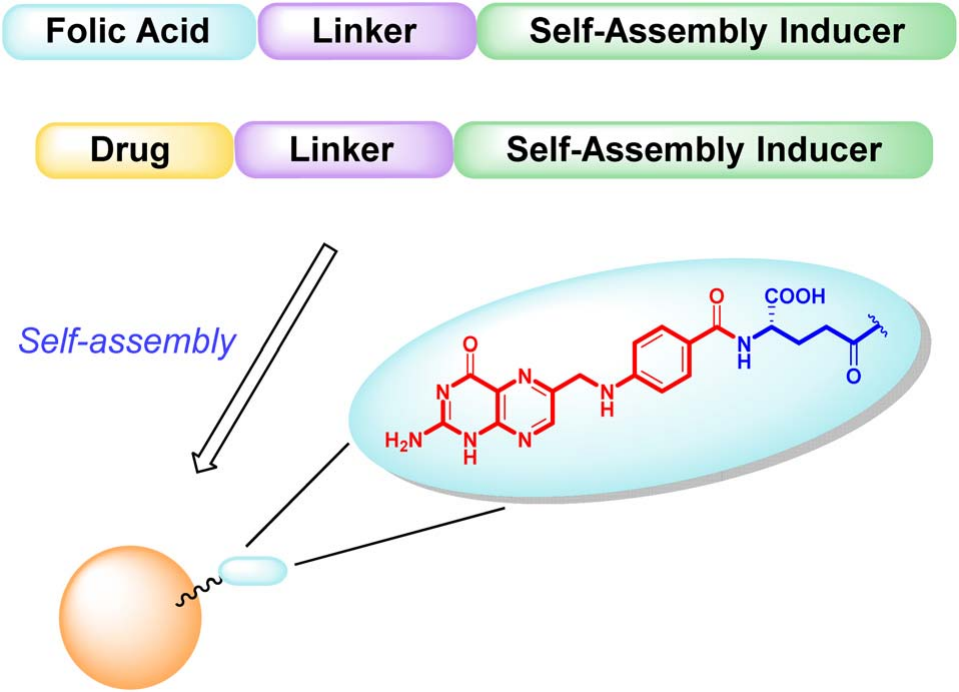
Schematic representation of targeted hetero-NPs, with folic acid highlighted in detail – pteroic acid in red, l-glutamic acid in blue.

The main objective of our work described here is to obtain hetero-NPs by the self-assembly of a conjugate based on folic acid (Fig. 1), to direct the hetero-NP to the tumor site, with another conjugate bearing the anticancer drug paclitaxel (PTX).

**Fig. 2:**
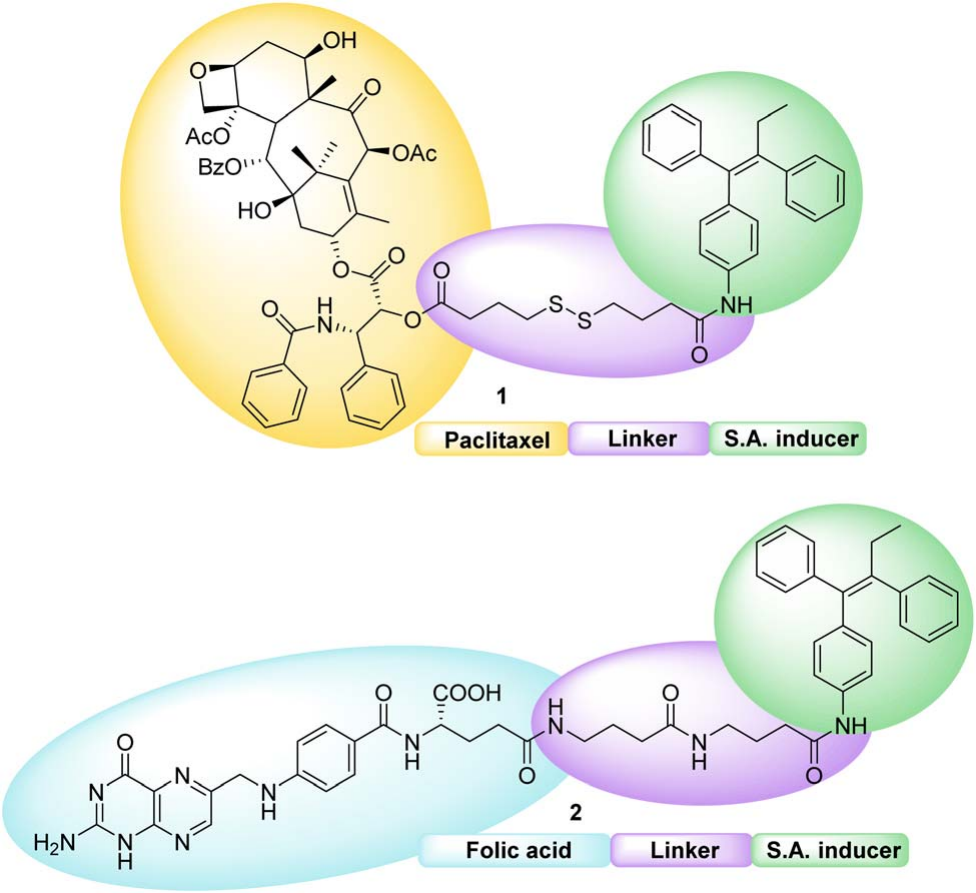
Chemical structure of paclitaxel (top, 1) and folic acid conjugates (bottom, 2).

Both conjugates, shown in Fig. 2, consist of an active fragment (either folic acid or paclitaxel) linked to a lipophilic self-assembly inducer through a specific linker. The linker itself is designed to be quite stable for the folic acid conjugate (bottom structure, 2), to prevent the NP from losing its targeting agent; conversely, a labile linker is used for the drug conjugate (top structure, 1, Fig. 2), to release paclitaxel at its site of action. The synthesis of both conjugates, their ability to form hetero-self-assembled NPs, and their cytotoxicity against tumor cells are here reported.

## Results and discussion

**Scheme 1:**
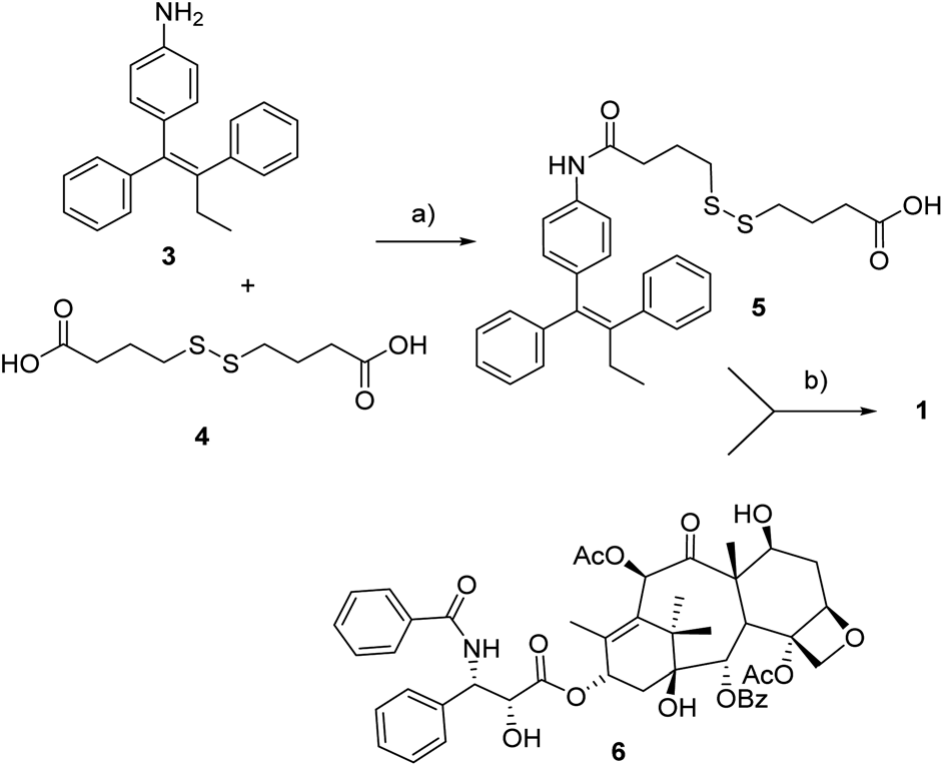
Synthesis of conjugate 1. a: DIPEA, HATU, dry THF, rt, 18 h, 30%, b: EDC·HCl, DMAP, rt, 22 h, 52%.

As to the preparation of drug conjugate 1, the linker-self-assembly inducer moiety 5 was synthesized according to a previously reported strategy (Scheme 1).^14^ At last, target compound 1 was obtained through the condensation between said moiety 5 with PTX 6 in presence of EDC and DMAP. The conjugation occurred with complete regioselectivity, although in moderate yield.

**Scheme 2:**
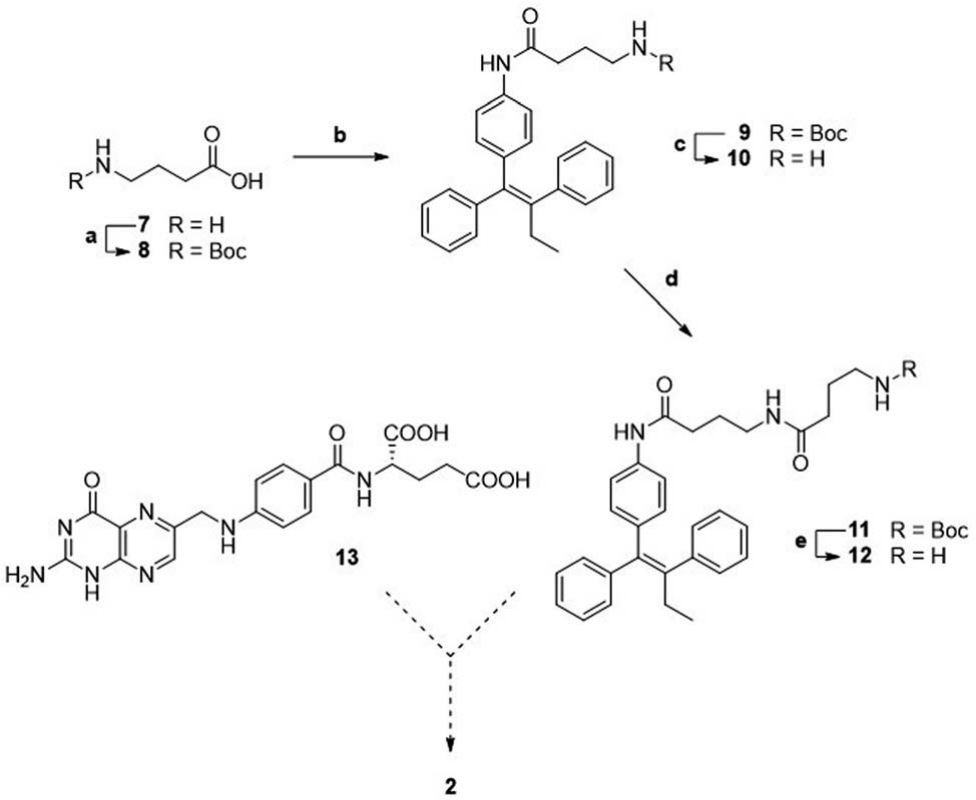
Synthesis of linker-self-assembly inducer 12. a: Boc_2_O, NaOH/diox, rt, 20 h, quant.; b: 3, HATU, DIPEA, dry THF, rt, 23 h, 94%; c: TFA, dry CH_2_Cl_2_, 0 °C to rt, 20 h, quant.; d: 8, HATU, DIPEA, dry THF, rt, 21 h, 73%; e: TFA, dry CH_2_Cl_2_, 0 °C to rt, 18 h, 90%.

A first attempted synthesis of the folic acid conjugate 2 resulted to be challenging (Scheme 2). The condensation of aniline 3 with *N*-Boc γ-aminobutyric acid 8 gave compound 9 that, after deprotection, was elongated with a second unit of *N*-Boc γ-aminobutyric acid, yielding Boc protected intermediate 11. After Boc removal, we obtained linker-self-assembly inducer conjugate 12 in overall good yields.

Unfortunately, despite multiple attempts performed in various reaction conditions, we could never obtain target folic acid conjugate 2 by direct condensation of folic acid 13 with intermediate 12. We attribute such failures to the scarce solubility of folic acid in organic solvents, leading to difficult reaction monitoring, to challenging recovery of the reaction products, and to unexpected side reactions.

**Scheme 3:**
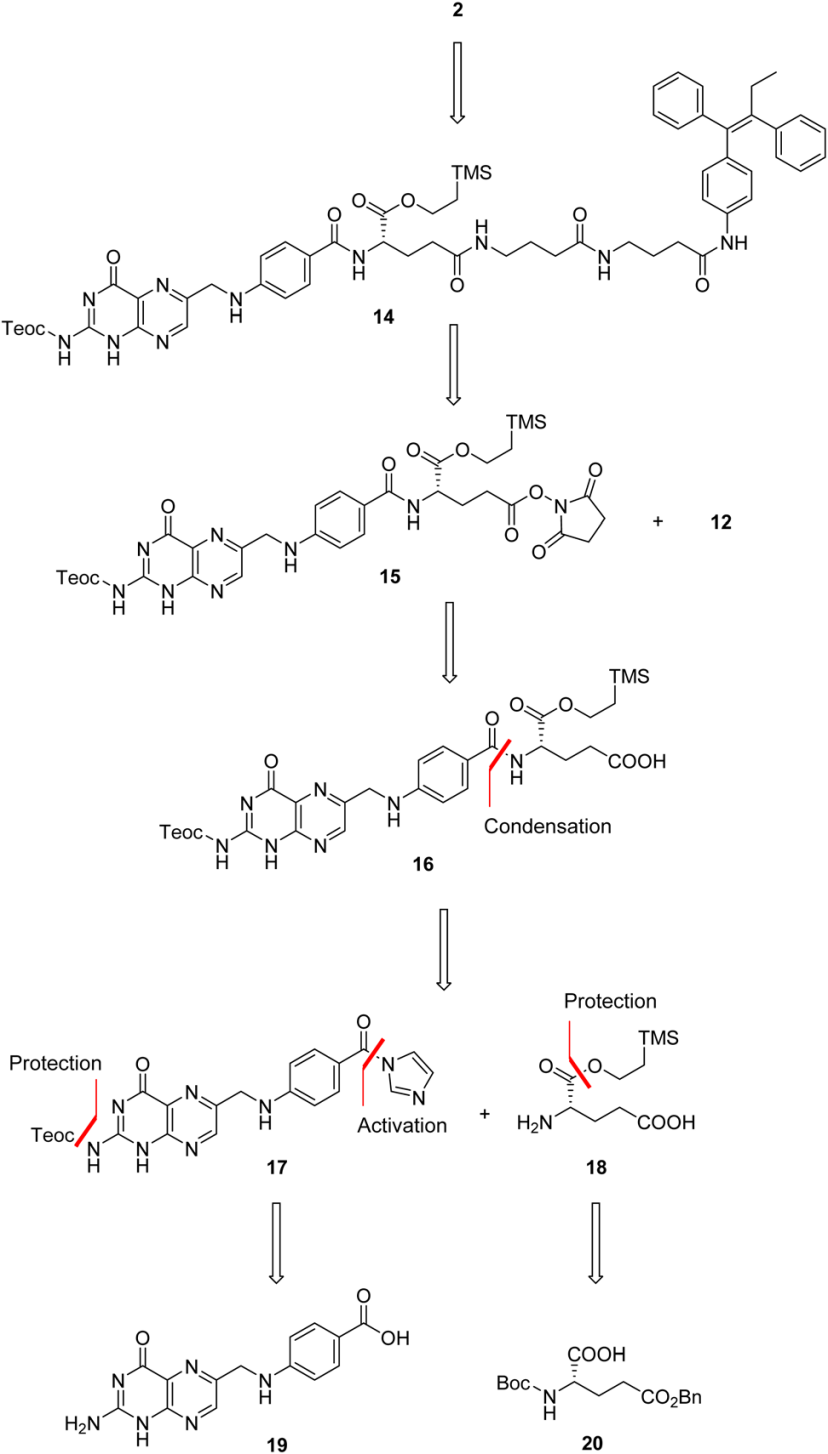
Retrosynthetic pathway for target folic acid conjugate 2.

We thus conceived a different retrosynthetic strategy that, even though complex, should have led us to target folic acid conjugate 2.^29^ A stepwise assembly entailed at first to build folic acid from pteroic acid and l-glutamic acid (see also Fig. 1), proceeding through a sequential protection/activation cascade. The retrosynthetic pathway is shown in Scheme 3, ensuring for the α-carboxylic acid to remain protected throughout the synthesis, avoiding its involvement in side reactions but also improving the solubility of intermediates due to lipophilic protecting groups.

In details, folic acid conjugate 2 should be obtained by deprotection of both amino and α-carboxyl groups of intermediate 14. Protected conjugate 14 should result from a coupling reaction between the linker-self-assembly inducer conjugate 12 and N,C_α_-protected and C_γ_-activated folic acid 15, to be obtained after *N*-hydroxysuccinimide (NHS)-activation of N,C_α_-protected folic acid 16. The latter could be achieved by condensing N-protected and C-activated pteroic acid 17 with C_α_-protected l-glutamic acid 18, which can be obtained through C_α_-protection and Boc and Bn-deprotection of commercial compound 20. Finally, intermediate 17 could be obtained by protecting the amine and activating the carboxyl group of commercial pteroic acid 19.

**Scheme 4:**
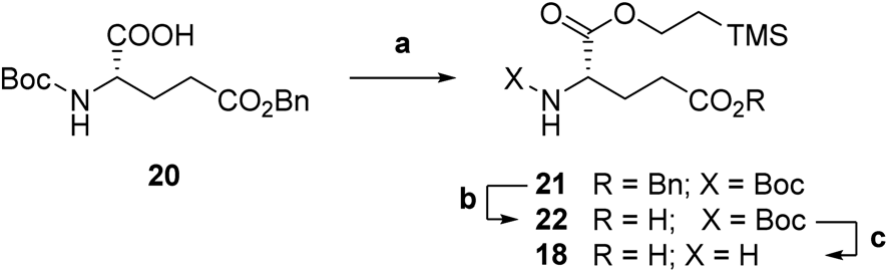
Synthesis of l-glutamic acid C_α_-TMSE ester 18. a: (1) CDI, dry CH_2_Cl_2_, 0 °C to rt, 1 h; (2) TMSEtOH, rt, 19 h, 54%; b: H_2_, Pd/C, dry EtOH, rt, 3 h, 91%; c: *p*TsOH·H_2_O, diox/H_2_O, 60 °C, 3 h, quant.

Accordingly, we targeted first a suitably C_α_-protected l-glutamic acid 18 to be coupled onto pteroic acid 19. Commercial Boc-l-glutamic acid 5-benzyl ester 20 was protected as a 2-(trimethylsilyl)ethyl (TMSE) ester (step a, Scheme 4) through a two-step, one pot procedure. Namely, compound 20 was first activated at its α-position with carbonyldiimidazole (CDI), and then treated with 2-(trimethylsilyl)ethanol to obtain fully protected diester 21. Then, selective deprotection of the C_γ_-benzyl ester in 21 by hydrogenolysis led to intermediate 22 (step b). Aminoester 18 was finally obtained as a *p*-toluensulfonate salt by *p*TsOH-mediated Boc deprotection (step c, Scheme 4) in overall good yields.

**Scheme 5:**
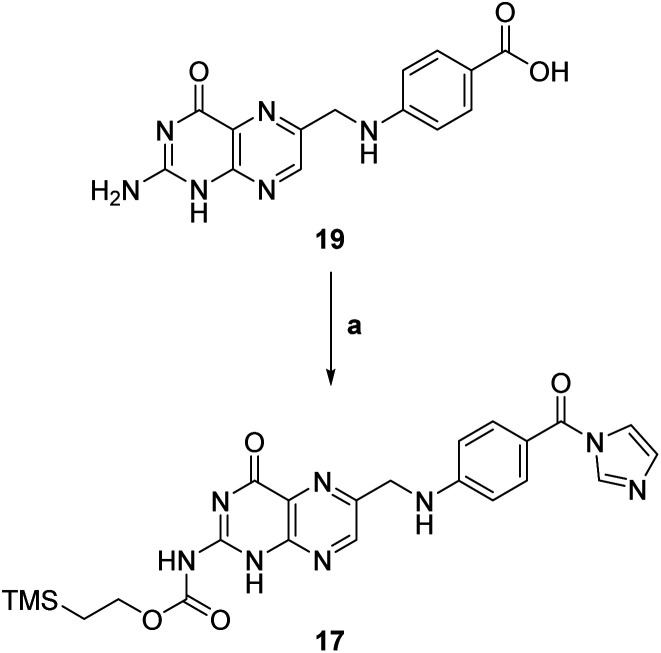
Synthesis of N-protected, C-activated pteroic acid 17. a: (1) CDI, dry DMSO, 50 °C, 5 h; (2) TMSEtOH, 50 °C, 21 h, 51%.

We focused then on the synthesis of suitably N-protected, C-activated pteroic acid derivative 17 in a two step, one pot procedure (step a, Scheme 5).

Treatment with CDI and TEA as a base in dry DMSO was meant to activate the carboxyl group as a carbonyl imidazole, and to convert the primary amine to a carbamate. Subsequently, 2-(trimethylsilyl)ethanol was to be added to carry out a nucleophilic acyl substitution on the carbamoyl imidazole, *de facto* providing a trimethylsilylethoxy carbamate (Teoc) protection for the amine group in compound 17.

The Teoc group was selected both to protect the amino group, decreasing the nitrogen's nucleophilicity and thus avoiding side reactions, and to improve the solubility of the resulting intermediate 17 in organic solvents due to its lipophilicity.

However, pteroic acid 19 showed limited solubility even in DMSO, complicating both reaction monitoring and purification. The first reaction attempts at rt were unsuccessful, as we only recovered starting material. To overcome poor solubility, following protocol published in literature,^29^ we heated the reaction to 50 °C, observing gradual solubilization of 19 and darkening of the reaction mixture. TLC monitoring after 26 hours showed a reaction product together with unreacted pteroic acid 19, but work-up and purification of the crude was affected by the poor solubility of 19 which diffused through both manual and automated flash chromatography. Thus, we recovered only 51% of pure N-protected, C-activated pteroic acid derivative 17 which, as expected, was now soluble in organic solvents and was thus used as such in the next reaction steps.

**Scheme 6:**
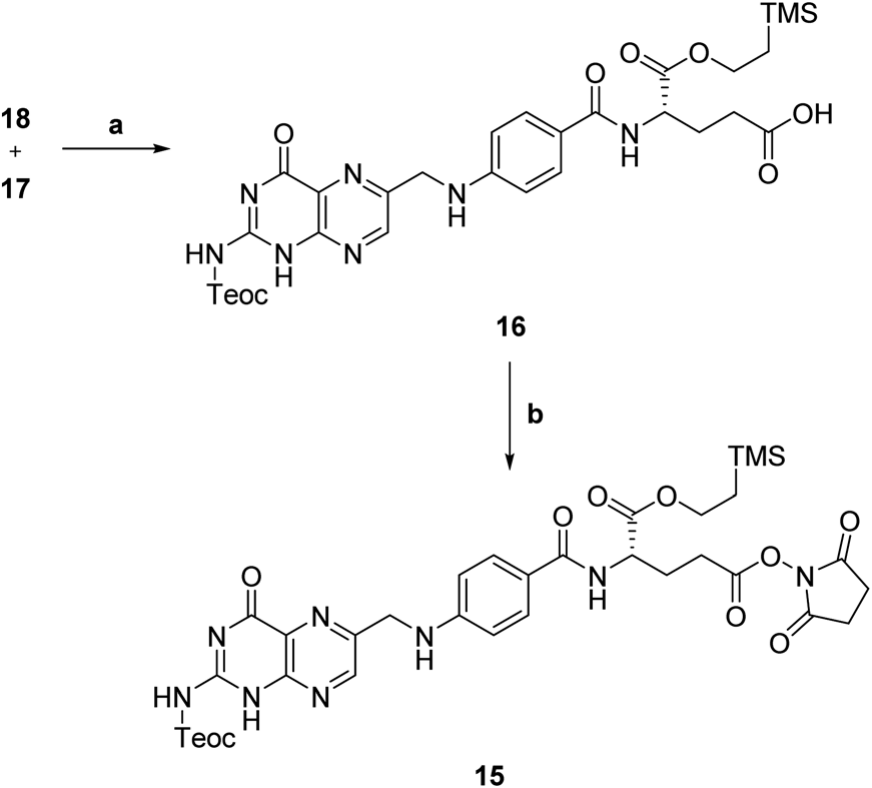
Synthesis of activated protected folic acid. a: MTBD, dry DMSO, rt, 21 h, 33%; b: NHS, EDC, dry DMF, rt, 19 h, 94%.

Then, N,C_α_-protected folic acid 16 was synthesized by coupling α-protected aminoester 18 with activated pteroic acid derivative 17 (step a, Scheme 6). The coupling was carried out in dry DMSO, in presence of a strong guanidine base. Protected folic acid 16 was obtained in poor, unoptimized yield, and then activated with NHS in standard conditions to yield the activated ester 15 (step b, Scheme 6) in excellent yield.

**Scheme 7:**
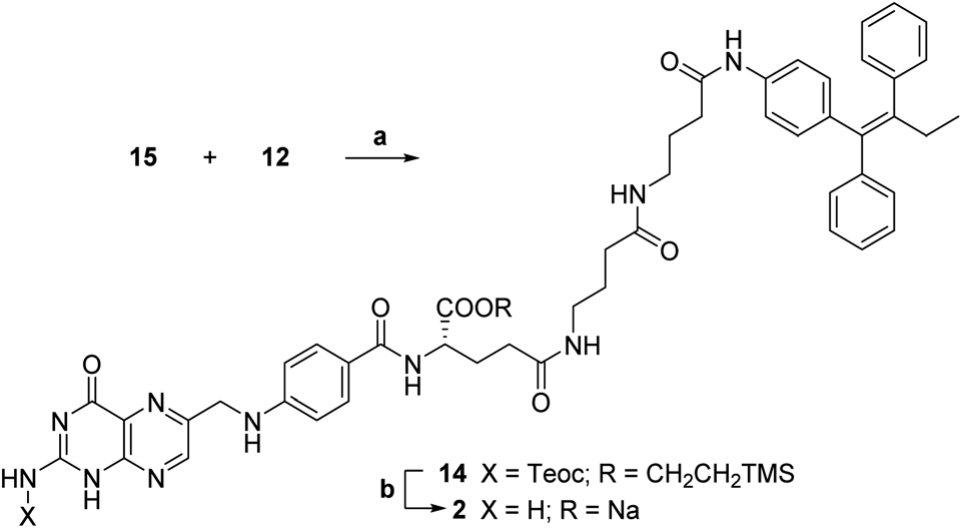
Synthesis of folic acid conjugate 2. a: TEA, dry DMSO, rt, 5 h, 40%; b: (1) 1 M TBAF, dry DMSO, rt, 19 h; (2) 0.2 M NaOAc, dry DMSO, rt, 10 min, 66%.

The key synthetic step of our strategy entailed the coupling of C_γ_-activated protected folic acid 15 with previously synthetized linker-self-assembly inducer conjugate 12 (step a, Scheme 7).

This basic condensation was successfully completed in dry DMSO, affording N,C-protected folic acid conjugate 14 in moderate yield after flash chromatography purification.

At last, the target folic acid conjugate 2 was obtained as a sodium salt in 66% yield by simultaneous deprotection of both the amino and the α-carboxyl group of compound 14 (step b, Scheme 7). Namely, treatment with tetrabutylammonium fluoride (TBAF) removed both silyl groups; then, the addition of a NaOAc solution caused the precipitation of the desired folic acid conjugate 2 as a sodium salt in good yield and purity without further purification.

Conjugates 1 and 2 were then used to form self-assembled hetero-NPs. Four nanosuspensions were prepared using standard solvent evaporation protocol,^30^ combining the conjugates in different proportions – namely, 0% (pure 1, hNP1), 5% (hNP2), 10% (hNP3), and 15% (hNP4) of conjugate 2.

**Fig. 3:**
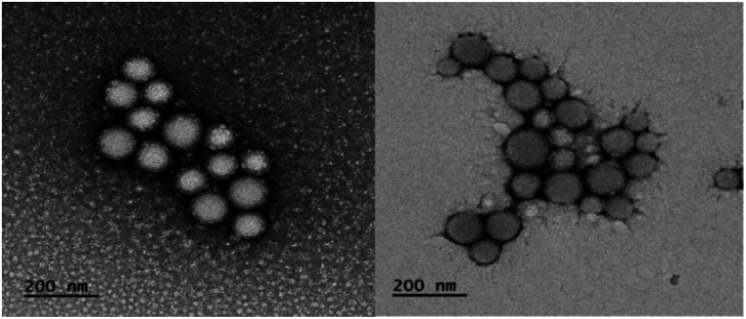
TEM micrographs of nanoparticles formed by self-assembly hNP2. All samples were stained with uranyl acetate solution.

Hetero-NPs were characterized by dynamic light scattering (DLS, Table 1) and transmission electron microscopy (TEM, Fig. 3).

**Table 1:** Polydispersity index, hydrodynamic diameter, and *Z*-potential of nanoformulations

	Polydispersity index (PI)	Hydrodynamic diameter – intensity distribution (nm)	Zeta potential (mV)
hNP1	0.221 ± 0.028	446.8 ± 58.8	−37.1 ± 0.61
hNP2	0.152 ± 0.044	325.8 ± 37.33	−35.7 ± 0.83
hNP3	0.188 ± 0.023	418.1 ± 36.57	−38.7 ± 0.61
hNP4	0.175 ± 0.024	364.9 ± 20.34	−39.0 ± 1.4

Self-assembly led to stable and monodisperse suspensions of all hetero-NPs, characterized by hydrodynamic diameters (HD) in the 320–450 nm range and a negative *Z*-potential (< −35.0 mV).

TEM images confirmed uniformity for each hetero-NP size and showed them to have a spherical shape (Fig. 3).

**Fig. 4:**
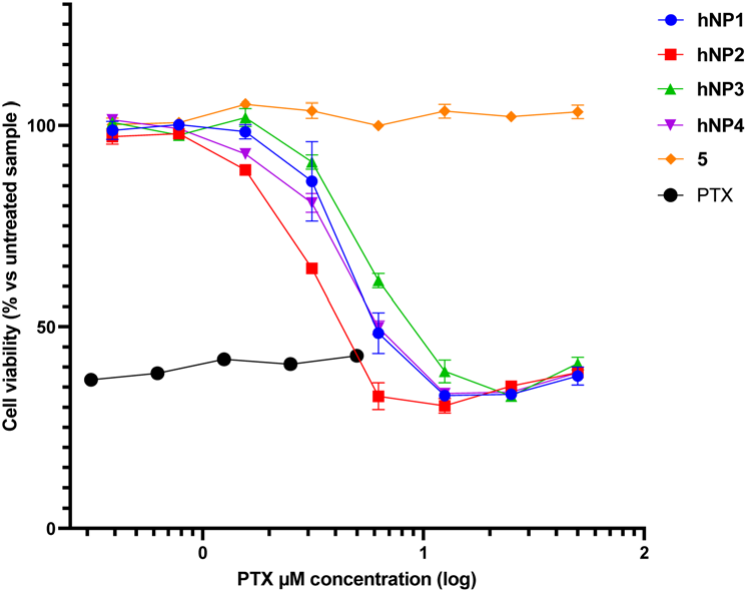
Cell viability for HeLa cells treated for 72 h with hNP1 to hNP4 at equivalent PTX concentrations (400 nM to 50 μM), and with self-assembly inducer 5 and free PTX 6 (312.5 nM to 5 μM) as control. Reported values are the mean ± SD (*n* = 3) normalized against untreated sample.

The effect on cell viability of each hetero-NP was evaluated by means of a colorimetric assay (MTS assay, Fig. 4).

For this purpose, cells were incubated with hetero-NPs at equivalent concentrations of PTX, ranging from 400 nM to 50 μM. In addition, free PTX 6 and self-assembly inducer 5 were used as controls, the former up to its solubility limit.

The results shown in Fig. 4 indicate that the self-assembly inducer 5 is not cytotoxic, while free PTX 6 and hetero-NPs hNP1–4 caused a significant reduction in cell viability (Table 2).

**Table 2:** IC_50_ obtained for hNP1–4

Sample	IC_50_ (μM)
hNP1	4.495 ± 0.466
hNP2	2.874 ± 0.109
hNP3	5.635 ± 0.035
hNP4	4.154 ± 0.088

Notably, hNP2 performed better than hNP1, made only of PTX conjugate 1, confirming that the inclusion of a targeting ligand may facilitate NPs' internalization.^31^ The reduced efficiency displayed by hNP3 and hNP4 suggests that an increased ligand density does not always correlate to an enhanced performance.^32^ It should also be noted that all hetero-NPs were less efficient than free PTX, as could be expected since PTX in hetero-NPs should be released only upon cytoplasmatic delivery and consequent reduction of the disulfide bridge.^4^

## Conclusions

We report here the synthesis and characterization of conjugates 1 and 2, overcoming significant hurdles related to solubility and purification. Such conjugates were self-assembled in different proportions, to obtain targeted hetero-NPs hNP1 to hNP4. Their analytical characterization confirms their nature as suitable nanoformulations for cancer therapy. Their biological evaluation indicates modest cytotoxic efficacy for folic acid-containing hetero-NPs, depending on the ratio between PTX and folic acid conjugates. This will encourage us to further investigate this kind of nanosystems, and to better evaluate the NPs uptake in other cell lines, expressing different levels of folate receptors.

Thus, by coupling a potent cytotoxic agent with an active targeting ligand in self-assembling hetero-NPs, we demonstrated a controlled release of PTX. We expect that, after further refinement, such approach will provide significant efficacy against cancer while mitigating the toxicity of the cytotoxic drug against non-cancer cells.

## Experimental

### Materials and methods

All reactions were carried out in oven-dried glassware and dry solvents under nitrogen atmosphere. Unless otherwise stated, all solvents were purchased from Sigma Aldrich and used without further purification. Substrates and reagents were purchased from Sigma Aldrich and used as received. Thin-layer chromatography (TLC) was performed on Merck precoated 60F_254_ plates. Reactions were monitored by TLC on silica gel, with detection by UV light (254 nm) or by charring with 1% permanganate solution. Flash chromatography was performed using silica gel (240–400 mesh, Merck). ^1^H-NMR spectra were recorded on Bruker DRX-400 and Bruker DRX-300 instruments and are reported relative to residual CDCl_3_. {^1^H}^13^C-NMR spectra were recorded on the same instruments (101 and 75 MHz) and are reported relative to CDCl_3_. Chemical shifts (*δ*) for proton and carbon resonances are quoted in parts per million (ppm) relative to tetramethylsilane (TMS), which was used as an internal standard. MS spectra were recorded using an electrospray ionization (ESI) technique on a Waters Micromass Q-Tof mass spectrometer.

### Synthetic procedures

#### Synthesis of 3 (ref. 33)

TiCl_4_ (3.85 g, 0.0203 mol) was added dropwise under nitrogen atmosphere to a stirred suspension of Zn dust (3.20 g, 0.0489 mol) in dry THF (43 mL) at −10 °C. The mixture was left stirring at 0 °C for 10 min until a blue coloration developed, then it was heated to reflux for 2 h. After cooling to 0 °C, a THF (57 mL) solution of 4-aminobenzophenone (1.00 g, 5.07 mmol) and propiophenone (749 mg, 5.58 mmol) was added. The reaction was left stirring at reflux for 2 h. The mixture was then poured into Na_2_CO_3_ 10% (72 mL) and was vigorously stirred at rt for 15 min. It was then filtered on celite, extracted with EtOAc (3 × 30 mL), washed with water (30 mL) and brine (30 mL). The collected organic phases were then dried over Na_2_SO_4_ and concentrated. The crude was purified by flash chromatography (silicagel, eluent mixture 8 : 2 v/v *n*-hex/EtOAc) to obtain pure 3 (1.12 g, 3.75 mol, 74% yield) as a beige solid.

##### Analytical characterization

^1^H-NMR (400 MHz, CDCl_3_): *δ* = 7.38–7.30 (m, 2H), 7.30–7.21 (m, 3H), 7.22–7.10 (m, 5H), 6.69 (d, *J* = 8.8 Hz, 2H), 6.42 (d, *J* = 8.8 Hz, 2H), 4.21 (bs, 2H), 2.44 (q, *J* = 7.6 Hz, 2H), 0.91 (t, *J* = 7.6 Hz, 3H).

^13^C-NMR (100 MHz, CDCl_3_): *δ* = 144.7, 144.0, 143.5, 141.4, 139.4, 134.7, 132.3 (2C), 130.4 (2C), 130.1 (2C), 128.6 (2C), 128.4, 127.0, 126.5 (2C), 115.2 (2C), 29.6, 14.0.

HRMS (ESI), *m*/*z*: calcd for C_22_H_21_N 299.1674, found 322.1580 (M + Na^+^).

#### Synthesis of 5 (ref. 14)

Solid HATU (210 mg, 0.551 mmol) and DIPEA (130 mg, 1.00 mmol) were added to a stirred solution of 4,4′-dithiodibutyric acid 4 (120 mg, 0.501 mmol) in dry THF (1.75 mL) under nitrogen atmosphere. The mixture was left stirring for 30 min, then 4-(1,2-diphenylbut-1-en-1-yl)aniline 3 (150 mg, 0.501 mmol) was added and the reaction mixture was left stirring at rt for 18 h. The solvent was evaporated under reduced pressure, EtOAc (3 mL) was added and the organic phase was washed with water (3 mL) and brine (3 mL). The collected organic phases were dried over Na_2_SO_4_ and concentrated under reduced pressure. The crude was purified by flash chromatography (silicagel, eluent mixture 6 : 4 v/v *n*-hex/EtOAc) to obtain pure 5 (0.052 g, 0.100 mmol, 20% yield) as a yellow oil.

##### Analytical characterization

^1^H-NMR (400 MHz, CDCl_3_): 7.45–7.07 (m, 12H), 6.88–6.78 (m, 2H), 2.84–2.70 (m, 4H), 2.58–2.36 (m, 6H), 2.15–1.98 (m, 4H), 0.95 (t, *J* = 7.7 Hz, 3H).

^13^C-NMR (100 MHz, CDCl_3_): *δ* = 178.3, 170.6, 143.4, 142.2, 139.2, 138.1, 137.9, 135.4, 131.4 (2C), 129.7 (2C), 129.5 (2C), 128.2 (2C), 127.9, 126.7, 126.2 (2C), 118.8 (2C), 37.8, 37.5, 35.4, 32.3, 29.0, 24.5, 24.0, 13.5.

HRMS (ESI), *m*/*z*: calcd for C_30_H_33_NO_3_S 519.1902, found 542.1836 (M + Na^+^).

#### Synthesis of 1

EDC·HCl (0.027.2 mg, 0.141 mmol) and DMAP (6.4 mg, 0.047 mmol) were added to a stirred solution of 5 (52.3 mg, 0.100 mmol) in dry CH_2_Cl_2_ (5 mL) under nitrogen atmosphere. Then 6 (56.9 mg, 0.0673 mmol) was added and the reaction mixture was left stirring at rt overnight. After reaction completion (TLC monitoring, eluent mixture 1 : 1 v/v *n*-hex/AcOEt), 1 M HCl (30 mL) was added, and the mixture was extracted with CH_2_Cl_2_ (5 × 10 mL). The collected organic phases were dried over Na_2_SO_4_ and concentrated under reduced pressure. The crude was purified by flash chromatography (silicagel, eluent mixture 1 : 1 v/v *n*-hex/EtOAc) to obtain pure target 1 (70.2 mg, 0.0348 mmol, 52% yield) as a white solid.

##### Analytical characterization

^1^H-NMR (400 MHz, CDCl_3_): *δ* = 8.17–8.10 (m, 2H), 7.78–7.68 (m, 2H), 7.65–7.56 (m, 1H), 7.55–7.46 (m, 3H), 7.46–7.29 (m, 10H), 7.25–7.03 (m, 9H), 6.83–6.75 (m, 2H), 6.32–6.28 (m, 1H), 6.28–6.19 (m, 1H), 6.03–5.93 (m, 1H), 5.68 (d, *J* = 7.0 Hz, 1H), 5.52 (d, *J* = 3.5 Hz, 1H), 4.97 (dd, *J* = 9.7, 2.6 Hz, 1H), 4.44 (dd, *J* = 10.9, 6.6 Hz, 1H), 4.31 (d, *J* = 8.4 Hz, 1H), 4.20 (d, *J* = 8.5 Hz, 1H), 3.81 (d, *J* = 6.9 Hz, 1H), 2.82–2.41 (m, 14H), 2.42–2.25 (m, 4H), 2.25–2.08 (m, 5H), 2.09–1.78 (m, 8H), 1.68 (s, 3H), 1.23–1.17 (m, 3H), 1.13 (s, 3H), 0.93 (t, *J* = 7.4 Hz, 3H).

^13^C-NMR (100 MHz, CDCl_3_): *δ* = 204.0, 172.2, 171.4, 170.5, 170.1, 170.0, 168.3, 167.2, 142.8, 142.3, 142.1, 140.0, 138.6, 137.4, 137.0, 136.0, 135.6, 133.8, 133.7, 133.0, 132.2, 131.6 (2C), 130.4 (2C), 129.8 (2C), 129.6 (2C), 129.3 (2C), 129.2 (2C), 128.9 (2C), 128.3 (2C), 128.0 (2C), 127.3, 127.2, 126.8, 126.73 (2C), 126.66 (2C), 118.9, 118.7, 84.6, 81.2, 79.3, 76.7, 75.7, 75.2, 74.2, 72.3, 72.0, 58.7, 55.2, 45.8, 43.3, 37.7, 37.2, 35.8, 35.7, 35.6, 32.1, 29.2, 26.9, 24.5, 24.1, 22.8, 22.2, 21.0, 15.0, 13.7, 9.8.

HRMS (ESI), *m*/*z*: calcd for C_77_H_82_N_2_O_16_S_2_ 1354.5106, found 1377.5018 (M + Na^+^).

#### Synthesis of 8 (ref. 34)

Solid Boc_2_O (3.18 g, 14.5 mmol) was added to a stirred solution of γ-aminobutyric acid 7 (1.00 g, 9.71 mmol) in NaOH 1 M (10 mL) and dioxane (2 mL). The reaction mixture was left stirring at rt overnight, and after reaction completion (TLC monitoring, eluent mixture 7 : 3 v/v *n*-hex/AcOEt) it was extracted with *n*-hexane (3 × 10 mL). The collected organic phases were washed with sat. NaHCO_3_ (2 × 8 mL); the combined aqueous phases were acidified with dil. HCl (5 mL) and extracted with EtOAc (7 × 20 mL). The reunited organic phases were then dried over Na_2_SO_4_ and concentrated under reduced pressure. The crude was purified by flash chromatography (silicagel, eluent mixture 7 : 3 v/v *n*-hex/EtOAc) to obtain pure target 8 (1.97 g, 9.68 mmol, quantitative yield) as a beige solid.

##### Analytical characterization

^1^H-NMR (400 MHz, CDCl_3_): *δ* = 10.88 (bs, 1H), 4.73 (bs, 1H), 3.27–3.04 (m, 2H), 2.38 (t, *J* = 7.2 Hz, 2H), 1.81 (quint, *J* = 7.0 Hz, 2H), 1.43 (s, 9H).

^13^C-NMR (100 MHz, CDCl_3_): *δ* 178.5, 156.3, 79.6, 39.9, 31.4, 28.5, 25.3 (3C).

HRMS (ESI), *m*/*z*: calcd for C_9_H_17_NO_4_ 203.1158, found 226.1058 (M + Na^+^).

#### Synthesis of 9

HATU (1.03 g, 2.71 mmol) and DIPEA (0.86 mL, 4.92 mmol) were added to a stirred solution of 8 (0.500 g, 2.46 mmol) in dry THF (39 mL) under nitrogen atmosphere. The reaction mixture was left stirring at rt for 30 min. 3 (0.742 g, 2.46 mmol) was then added, and the solution was stirred at rt overnight. After reaction completion (TLC monitoring, eluent mixture 1 : 1 v/v *n*-hex/AcOEt) the solvent was evaporated under reduced pressure, 1 M HCl (15 mL) was added and the aqueous phase was extracted with CH_2_Cl_2_ (3 × 15 mL). The collected organic phases were dried over Na_2_SO_4_ and concentrated under reduced pressure. The crude was purified by flash chromatography (silicagel, eluent mixture 1 : 1 v/v *n*-hex/EtOAc) to obtain pure target 9 (1.12 g, 2.31 mmol, 94% yield) as a beige solid.

##### Analytical characterization

^1^H-NMR (400 MHz, CDCl_3_): *δ* = 8.45 (bs, 1H), 7.32–7.23 (m, 2H), 7.32–7.23 (m, 5H), 7.22–7.10 (m, 5H), 6.84 (d, *J* = 8.5 Hz, 2H), 4.76 (bs, 1H), 3.21 (q, *J* = 6.3 Hz, 2H), 2.51–2.46 (m, 2H), 2.31 (t, *J* = 6.6 Hz, 2H), 1.88–1.78 (m, 2H), 1.44 (s, 9H), 0.95 (t, *J* = 7.4 Hz, 3H).

^13^C-NMR (100 MHz, CDCl_3_): *δ* = 170.8, 150.4, 143.7, 142.3, 138.8, 137.6, 137.1, 131.9, 131.5 (2C), 129.8 (2C), 129.6 (2C), 128.2 (2C), 128.0 (2C), 126.7, 126.3, 118.7 (2C), 79.9, 39.4, 34.8, 29.2, 28.5 (3C), 23.2, 13.7.

HRMS (ESI), *m*/*z*: calcd for C_31_H_36_N_2_O_3_ 484.2726, found 506.2629 (M + Na^+^).

#### Synthesis of 10

TFA (3.39 mL, 0.044 mol) was added to a stirred solution of 9 (0.980 g, 2.02 mmol) in dry CH_2_Cl_2_ (68 mL) at 0 °C, under nitrogen atmosphere. The reaction mixture was then left stirring at rt overnight, and after reaction completion (TLC monitoring, eluent mixture 1 : 1 v/v *n*-hex/AcOEt) the solvent was evaporated under reduced pressure to obtain target trifluoroacetate 10 (1.01 g, 2.00 mmol, quant. yield) as a beige solid, without further purification.

##### Analytical characterization

^1^H-NMR (400 MHz, CDCl_3_): *δ* = 7.88 (bs, 1H), 7.38–7.30 (m, 2H), 7.27–7.17 (m, 5H), 7.14–7.01 (m, 5H), 6.82 (d, *J* = 8.5 Hz, 2H), 3.88 (bs, 3H), 3.11–2.86 (m, 2H), 2.45 (m, 4H), 2.00–1.77 (m, 2H), 0.91 (t, *J* = 7.4 Hz, 3H).

^13^C-NMR (100 MHz, CDCl_3_): *δ* = 171.6, 143.2, 142.7, 142.0, 140.2, 137.9, 134.4, 131.3 (2C), 129.6 (2C), 129.4 (2C), 128.2 (2C), 127.9 (2C), 126.8, 126.3, 119.6 (2C), 39.7, 34.1, 29.1, 22.6, 13.5.

HRMS (ESI), *m*/*z*: calcd for C_28_H_29_F_3_N_2_O_2_ 482.2181, found 407.2110 (M-TFA + Na^+^).

#### Synthesis of 11

HATU (1.82 g, 4.79 mmol) and DIPEA (1.24 g, 9.58 mmol) were added to a stirred solution of N-protected γ-aminobutyric acid 8 (0.491 g, 2.40 mmol) in dry THF (40 mL), and the reaction mixture was left stirring at rt for 30 min under nitrogen atmosphere. A solution of 10 (1.20 g, 2.40 mmol) in dry THF (5 mL) was then added, and the reaction was stirred at rt overnight until completion (TLC monitoring, eluent mixture 2 : 8 v/v *n*-hex/AcOEt). The solvent was evaporated under reduced pressure and the crude was purified by flash chromatography (silicagel, eluent mixture 2 : 8 v/v *n*-hex/EtOAc) to obtain pure target 11 (1.00 g, 1.75 mmol, 73% yield) as a beige solid.

##### Analytical characterization

^1^H-NMR (400 MHz, CDCl_3_): *δ* = 7.35–7.31 (m, 2H), 7.27–7.22 (m, 5H), 7.20–7.09 (m, 5H), 6.80 (d, *J* = 8.6 Hz, 2H), 4.72 (bs, 1H), 3.33 (q, *J* = 6.1 Hz, 2H), 3.14 (q, *J* = 6.4 Hz, 2H), 2.47 (q, *J* = 7.4 Hz, 2H), 2.30 (t, *J* = 6.6 Hz, 2H), 2.20 (t, *J* = 6.7, 2H), 1.90–1.81 (m, 2H), 1.81–1.72 (m, 2H), 1.42 (s, 9H), 0.93 (t, *J* = 7.4 Hz, 3H).

^13^C-NMR (100 MHz, CDCl_3_): *δ* = 173.8, 171.1, 159.9, 143.7, 142.4, 142.0, 138.8, 138.5, 136.3, 131.4 (2C), 129.8 (2C), 129.7 (2C), 128.2 (2C), 128.0 (2C), 126.7, 126.3, 118.8 (2C), 79.8, 39.6, 38.6, 34.9, 33.7, 29.2, 28.6 (3C), 26.8, 26.7, 13.7.

HRMS (ESI), *m*/*z*: calcd for C_35_H_43_N_3_O_4_ 569.3254, found 592.3165 (M + Na^+^).

#### Synthesis of 12

TFA (4.47 mL, 0.0584 mol) was added to a stirred solution of 11 (1.66 g, 2.92 mmol) in dry CH_2_Cl_2_ (98 mL) at 0 °C under nitrogen atmosphere. The reaction mixture was then left stirring at rt overnight. After reaction completion (TLC monitoring, eluent mixture 1 : 1 v/v *n*-hex/AcOEt), sat. NaHCO_3_ was added (50 mL) and the aqueous phase was extracted with CH_2_Cl_2_ (50 mL) and EtOAc (2 × 50 mL). The collected organic phases were dried over Na_2_SO_4_ and concentrated under reduced pressure. The crude was purified by flash chromatography (silicagel, eluent mixture 8 : 2 v/v CH_2_Cl_2_/MeOH) to obtain pure target 12 (1.23 g, 2.63 mmol, 90% yield) as a beige solid.

##### Analytical characterization

^1^H-NMR (400 MHz, CDCl_3_): *δ* = 7.33 (m, 2H), 7.28–6.92 (m, 10H), 6.84 (d, *J* = 8.0 Hz, 2H), 4.45 (bs, 3H), 3.27–2.95 (m, 2H), 2.84–2.76 (m, 2H), 2.62–2.42 (m, 2H), 2.29–1.99 (m, 4H), 1.95–1.75 (m, 2H), 1.75–1.53 (m, 2H), 0.96 (t, *J* = 7.2 Hz, 3H).

^13^C-NMR (100 MHz, CDCl_3_): *δ* = 173.9, 171.3, 143.6, 142.4, 141.6, 138.8, 138.4, 136.4, 131.5 (2C), 130.0 (2C), 129.3 (2C), 128.2 (2C), 127.9 (2C), 126.7, 126.2, 118.7 (2C), 40.2, 38.6, 34.3, 33.4, 29.2, 27.5, 26.8, 13.7.

HRMS (ESI), *m*/*z*: calcd for C_32_H_36_F_3_N_3_O_3_ 567.2709, found 492.2634 (M-TFA + Na^+^).

#### Synthesis of 21 (ref. 35)

Solid CDI (0.982 g, 6.07 mmol) was added to a stirred solution of 20 (2.05 g, 6.07 mmol) in dry CH_2_Cl_2_ (28 mL) at 0 °C, and the mixture was left stirring at rt for 1 h under nitrogen atmosphere. 2-(Trimethylsilyl)ethanol (0.871 mL, 6.07 mmol) was then added and the reaction mixture was stirred at rt overnight until reaction completion (TLC monitoring, eluent mixture 3 : 1 v/v *n*-hex/AcOEt). Then it was washed with water (15 mL), and the collected organic phases were dried over Na_2_SO_4_ and concentrated under reduced pressure. The crude was purified by flash chromatography (silicagel, eluent mixture 3 : 1 v/v *n*-hex/EtOAc) to obtain pure target 21 (1.42 g, 3.28 mmol, 54% yield) as a white solid.

##### Analytical characterization

^1^H-NMR (400 MHz, CDCl_3_): *δ* = 7.41–7.28 (m, 5H), 5.12 (s, 2H), 5.09 (bs, 1H), 4.34–4.26 (m, 1H), 4.24–4.18 (m, 2H), 2.56–2.36 (m, 2H), 2.29–2.12 (m, 1H), 2.02–1.89 (m, 1H), 1.43 (s, 9H), 1.02–0.98 (m, 2H), 0.04 (s, 9H).

^13^C-NMR (100 MHz, CDCl_3_): *δ* = 172.5, 172.1, 156.0, 135.8, 128.5 (2C), 128.3 (2C), 128.0, 79.4, 66.0, 63.4, 52.9, 30.6, 28.3 (3C), 27.3, 17.8, −1.6 (3C).

HRMS (ESI), *m*/*z*: calcd for C_22_H_35_NO_6_Si 437.2234, found 460.2139 (M + Na^+^).

#### Synthesis of 22 (ref. 29)

10% Pd/C (0.132 g, 1.24 mmol) was added to a stirred solution of 21 (0.965 g, 2.21 mmol) in dry EtOH (10 mL). The reaction mixture was left stirring under H_2_ atmosphere at rt for 3 h, until reaction completion (TLC monitoring, eluent mixture 1 : 1 v/v *n*-hex/AcOEt). The suspension was then filtered on celite and then concentrated under reduced pressure. The crude was purified by flash chromatography (silicagel, eluent mixture 9 : 1 v/v *n*-hex/EtOAc) to obtain pure target 22 (0.705 g, 2.01 mmol, 91% yield) as a white solid.

##### Analytical characterization

^1^H-NMR (400 MHz, CDCl_3_): *δ* = 5.17 (m, 1H), 4.42–4.27 (m, 1H), 4.24–4.20 (m, 2H), 2.49–2.40 (m, 2H), 2.23–2.17 (m, 1H), 1.96–1.89 (m, 1H), 1.46 (s, 9H), 1.04–0.99 (m, 2H), 0.06 (s, 9H).

^13^C-NMR (100 MHz, CDCl_3_): *δ* = 177.9, 172.1, 155.4, 80.1, 64.0, 52.8, 30.1, 28.3 (3C), 27.8, 17.4, −1.5 (3C).

HRMS (ESI), *m*/*z*: calcd for C_15_H_29_NO_6_Si 347.1764, found 370.1670 (M + Na^+^).

#### Synthesis of 18 (ref. 29)

*p*TosOH (0.550 g, 2.89 mmol) was added to a stirred solution of 22 (0.669 g, 1.93 mmol) in a 1 : 3 water–dioxane mixture (4.6 mL). The reaction mixture was left stirring at 60 °C for 3 h until reaction completion (TLC monitoring, eluent mixture 8 : 2 v/v CH_2_Cl_2_/MeOH). The reaction was then quenched with a NaOH solution (2 mL), the solvent was evaporated under reduced pressure and the crude was purified by flash chromatography (silicagel, eluent mixture 8 : 2 v/v CH_2_Cl_2_/MeOH) to obtain pure target 18 (0.447 g, 1.90 mmol, quant. yield) as a white solid.

##### Analytical characterization

^1^H-NMR (400 MHz, CDCl_3_): *δ* = 4.27–4.22 (m, 3H), 2.65–2.61 (m, 2H), 2.33–2.26 (m, 2H), 1.07–0.94 (m, 2H), 0.05 (s, 9H).

^13^C-NMR (100 MHz, CDCl_3_): *δ* = 174.6, 174.1, 62.3, 53.2, 30.8, 29.1, 16.9, −1.4 (3C).

HRMS (ESI), *m*/*z*: calcd for C_10_H_21_NO_4_Si 247.1240, found 270.1143 (M + Na^+^).

#### Synthesis of 17 (ref. 29)

TEA (0.534 mL, 3.84 mmol) and CDI (0.623 g, 3.84 mmol) were added under nitrogen atmosphere to a stirred solution of pteroic acid 19 (0.200 g, 0.640 mmol) in dry DMSO (6.4 mL), heating the flask with a heat gun between each addition, until a darker coloration developed. The reaction mixture was left stirring at 50 °C for 4 h. 2-(Trimethylsilyl)ethanol (1.10 mL, 7.68 mmol) was then added, and the reaction mixture was stirred at 50 °C overnight until reaction completion (TLC monitoring, eluent mixture 9 : 1 v/v CHCl_3_/MeOH). The mixture was poured in a stirred suspension of water (44 mL), AcOH (1.28 mL) and Et_2_O (11.6 mL). The resulting precipitate was filtered, dried and purified by flash chromatography (silicagel, eluent mixture 9 : 1 v/v CHCl_3_/MeOH) to obtain pure target 17 (0.165 g, 0.326 mmol, 51% yield) as a yellow solid.

##### Analytical characterization

^1^H-NMR (400 MHz, DMSO-d_6_): *δ* = 11.76 (bs, 1H), 11.68 (bs, 1H), 8.88 (s, 1H), 8.14 (s, 1H), 7.66 (t, *J* = 6.1 Hz, 1H), 7.61 (d, *J* = 8.0 Hz, 2H), 7.61–7.58 (m, 1H), 7.09 (s, 1H), 6.77 (d, *J* = 8.8 Hz, 2H), 4.66 (d, *J* = 6.1 Hz, 2H), 4.34–4.25 (m, 2H), 1.09–1.00 (m, 2H), 0.05 (s, 9H).

^13^C-NMR (100 MHz, DMSO-d_6_): *δ* = 164.9, 159.4, 159.2, 154.9, 154.6, 153.0, 151.4, 149.2, 138.0, 132.7 (2C), 130.1, 129.7, 118.7, 117.6, 111.7 (2C), 64.6, 45.6, 17.0, −1.5 (3C).

HRMS (ESI), *m*/*z*: calcd for C_23_H_26_N_8_O_4_Si 506.1846, found 529.1760 (M + Na^+^).

#### Synthesis of 16 (ref. 36)

MTBD (0.116 mL, 0.809 mmol) was added under nitrogen atmosphere to a stirred solution of 17 (0.102 g, 0.202 mmol) and 18 (0.100 g, 0.404 mmol) in dry DMSO (1 mL). The reaction was left stirring at rt overnight. The mixture was then poured in a mixture of 1 M AcOH (54 mL), MeOH (23 mL) and CHCl_3_ (54 mL). After being extracted, the organic phase was washed with a 1 : 1 v/v mixture of 1 M AcOH/MeOH (36 mL) and with 2 : 1 v/v water/MeOH (2 × 54 mL); then it was dried over Na_2_SO_4_ and concentrated under reduced pressure. The crude was purified by flash chromatography (silicagel, eluent mixture 17 : 1 : 2 : 0.08 v/v CHCl_3_/MeOH/EtOAc/AcOH) to obtain pure 16 (46.2 mg, 0.0667 mmol, 33% yield) as a yellow solid.

##### Analytical characterization

^1^H-NMR (400 MHz, CDCl_3_): *δ* = 11.91 (bs, 1H), 8.75 (s, 1H), 7.55 (d, *J* = 7.9 Hz, 2H), 7.12 (s, 1H), 6.48 (s, 2H), 4.78–4.72 (m, 1H), 4.55 (s, 1H), 4.37 (t, *J* = 8.7 Hz, 3H), 4.23 (t, *J* = 8.4 Hz, 3H), 2.53–2.48 (m, 2H), 2.40–2.27 (m, 1H), 2.18–2.14 (m, 1H), 1.12 (t, *J* = 8.8 Hz, 3H), 1.02 (t, *J* = 8.7 Hz, 3H), 0.08 (s, 9H), 0.03 (s, 9H).

^13^C-NMR (100 MHz, CDCl_3_): *δ* = 173.6, 172.1, 166.2, 159.3, 154.8, 154.4, 151.9, 150.5, 149.0, 148.9, 129.8 (2C), 128.9, 121.2, 111.1 (2C), 64.6, 62.4, 52.0, 46.0, 30.2, 25.8, 17.1, 16.8, −1.43 (3C), −1.45 (3C).

HRMS (ESI), *m*/*z*: calcd for C_30_H_43_N_7_O_8_Si_2_ 685.2712, found 708.2618 (M + Na^+^).

#### Synthesis of 15 (ref. 36)

NHS (9.2 mg, 0.084 mmol) and EDC·HCl (13.4 mg, 0.067 mmol) were added to a stirred solution of 16 (45.8 mg, 0.067 mmol) in dry DMF (0.940 mL) under nitrogen atmosphere. The reaction mixture was left stirring at rt overnight, then more NHS (0.5 eq.) and EDC·HCl (0.5 eq.) were added and stirring was continued for 4 h until reaction completion (TLC monitoring, eluent mixture 9 : 1 v/v CH_2_Cl_2_/MeOH). The reaction mixture was then poured in water (5 mL) and the formed precipitate was filtered and dried to obtain pure 15 (49.4 mg, 0.0630 mmol, 94% yield) as a yellow solid.

##### Analytical characterization

^1^H-NMR (400 MHz, DMSO-d_6_): *δ* = 11.72 (bs, 1H), 8.84 (s, 1H), 8.31 (d, *J* = 7.4 Hz, 1H), 7.66 (d, *J* = 8.3 Hz, 2H), 7.04 (t, *J* = 6.2 Hz, 1H), 6.66 (d, *J* = 8.3 Hz, 2H), 4.59 (d, *J* = 6.1 Hz, 2H), 4.47–4.37 (m, 1H), 4.29 (t, *J* = 8.5 Hz, 2H), 4.13 (t, *J* = 8.3 Hz, 2H), 2.87–2.74 (m, 6H), 2.14–2.05 (m, 2H), 1.05 (t, *J* = 8.5 Hz, 2H), 0.93 (t, *J* = 8.4 Hz, 2H), 0.06 (s, 9H), 0.00 (s, 9H).

^13^C-NMR (100 MHz, DMSO-d_6_): *δ* = 171.1, 170.0, 169.2 (2C), 169.0, 166.3, 162.4, 154.8, 154.4, 151.8, 150.5, 149.1, 148.9, 129.8 (2C), 128.8, 121.1, 111.3 (2C), 64.7, 62.4, 51.9, 45.9, 30.4, 25.8, 17.6 (2C), 16.8, −1.43 (3C), −1.40 (3C).

HRMS (ESI), *m*/*z*: calcd for C_34_H_46_N_8_O_10_Si_2_ 782.2875, found 805.2788 (M + Na^+^).

#### Synthesis of 14

TEA (41 μL, 0.308 mmol) was added under nitrogen atmosphere to a stirred solution of 15 (0.133 g, 0.169 mmol) and 12 (0.0722 g, 0.154 mmol) in dry DMSO (3.1 mL), and the reaction mixture was left stirring at rt for 5 h until reaction completion (TLC monitoring, eluent mixture 9 : 1 v/v CH_2_Cl_2_/MeOH). Water (4.5 mL) was added until formation of a precipitate, which was centrifuged, washed with water (3 × 5 mL) and dried. The crude was purified by flash chromatography (silicagel, eluent mixture 96 : 4 v/v CH_2_Cl_2_/MeOH) to obtain pure target 14 (70.8 mg, 0.0616 mmol, 40% yield) as a light brown solid.

##### Analytical characterization

^1^H-NMR (400 MHz, DMSO-d_6_): *δ* = 9.77 (s, 1H), 8.70 (s, 1H), 8.29 (d, *J* = 7.3 Hz, 1H), 7.81 (dt, *J* = 11.9, 5.5 Hz, 2H), 7.65–7.57 (m, 3H), 7.39–7.35 (m, 2H), 7.32–7.17 (m, 7H), 7.14–7.08 (m, 3H), 7.03–6.92 (m, 2H), 6.73–6.71 (m, 2H), 6.65 (d, *J* = 8.4 Hz, 2H), 4.52 (d, *J* = 5.9 Hz, 2H), 4.31–4.26 (m, 1H), 4.19–4.08 (m, 4H), 3.09–2.95 (m, 4H), 2.42–2.32 (m, 2H), 2.23–1.99 (m, 6H), 1.92–1.86 (m, 1H), 1.67–1.54 (m, 4H), 1.00–0.88 (m, 4), 0.87–0.82 (m, 3H), 0.05 (s, 9H), 0.02 (s, 9H).

^13^C-NMR (100 MHz, DMSO-d_6_): *δ* = 174.0, 173.2, 172.9, 171.6, 167.2, 162.4, 153.3, 151.9, 151.2, 149.7, 149.6, 145.6, 142.2, 140.7, 138.6, 137.4, 136.1, 132.4, 131.6, 130.8, 130.2 (2C), 128.6 (2C), 128.5 (2C), 128.4 (2C), 127.6 (2C), 127.5, 123.6, 123.1 (2C), 117.5 (2C), 112.6 (2C), 63.7, 63.6, 52.7, 42.1, 39.9, 39.8, 34.3, 33.9, 32.2, 28.7, 27.3, 24.4, 24.3, 17.9, 17.8, 14.4, −1.4 (3C), −1.4 (3C).

HRMS (ESI), *m*/*z*: calcd for C_60_H_76_N_10_O_9_Si_2_ 1136.5335, found 1159.5248 (M + Na^+^).

#### Synthesis of 2

1 M TBAF (0.562 mL, 0.562 mmol) was added to a stirred solution of 14 (63.8 mg, 0.0562 mmol) in dry DMSO (0.56 mL) under nitrogen atmosphere, and the reaction mixture was left stirring at rt for 19 h. AcOH (0.703 mL) was then added and the mixture was poured in a 4 : 1 v/v mixture of CHCl_3_/EtOAc (14.05 mL) in a failed attempt to precipitate the product. Solvents were then evaporated under reduced pressure and the solid residue was dissolved in a 1 : 1 v/v mixture of EtOH/MeOH (3.93 mL). 0.2 M NaOAc in MeOH (0.90 mL) was then added, observing the formation of a precipitate, which was then centrifuged, washed with a 1 : 1 v/v mixture of MeOH/EtOH (3 × 5 mL) and dried to obtain pure target 2 (34.2 mg, 0.0371 mmol, 66% yield) as a yellow solid.

##### Analytical characterization

^1^H-NMR (400 MHz, DMSO-d_6_): *δ* = 9.94 (s, 1H), 8.98 (s, 1H), 8.64 (s, 1H), 7.88 (bs, 2H), 7.64–7.58 (m, 3H), 7.38 (t, *J* = 7.4 Hz, 3H), 7.28 (d, *J* = 8.4 Hz, 2H), 7.22–7.16 (m, 4H), 7.12 (d, *J* = 7.8 Hz, 3H), 6.86 (d, *J* = 5.8 Hz, 2H), 6.73 (d, *J* = 7.9 Hz, 2H), 6.67 (d, *J* = 8.1 Hz, 1H), 4.49 (d, *J* = 5.9 Hz, 2H), 4.12 (bs, 2H), 3.04–3.00 (m, 4H), 2.41–2.35 (m, 2H), 2.27–2.02 (m, 6H), 1.76–1.69 (m, 1H), 1.68–1.56 (m, 4H), 0.86 (t, *J* = 7.0 Hz, 3H).

^13^C-NMR (100 MHz, DMSO-d_6_): *δ* = 178.8, 178.7, 174.7, 173.4, 171.7, 167.4, 162.9, 159.6, 152.1, 149.9, 145.8, 142.4, 141.2, 139.5, 137.6, 136.1, 133.0, 132.3 (2C), 131.2 (2C), 130.3 (2C), 129.0 (2C), 128.8 (2C), 128.6, 127.9, 127.7 (2C), 122.7, 122.4, 117.6 (2C), 112.7 (2C), 53.0, 41.8, 40.2, 40.0, 34.3, 34.0, 32.0, 28.8, 27.1, 23.5 (2C), 14.0.

HRMS (ESI), *m*/*z*: calcd for C_49_H_52_N_10_O_7_ 892.402, found 915.392 (M + Na^+^).

### Nanoparticles assembly

The two conjugates 1 and 2 are mixed with the desired ratio (0%, 5%, 10% and 15% mol, for a total of 4.0 mg). To achieve better results, while compound 1 was weighted and added as a powder, compound 2 was used as a 1 mg mL^−1^ solution 2% DMSO in THF. THF is added to the mixture, to reach the total volume of 1 mL. The resulting solution was added dropwise to a round bottom flask containing MilliQ grade distilled water (2 mL) under magnetic stirring (500 rpm). The resulting suspension was stirred for 5 min, then THF was thoroughly evaporated under reduced pressure, obtaining an opalescent suspension of hetero-NPs (2 mL, 2 mg mL^−1^).

### Nanoparticles characterization

#### Dynamic light scattering (DLS)

The hydrodynamic diameter and *ζ*-potential for hNP1 to hNP4 were analyzed on a Zetasizer Nano ZS ZEN3600 (Malvern Panalytical, Malvern, Worcestershire, UK) operating at a light source wavelength of 633 nm and a fixed scattering angle of 173°. For both DLS and *ζ*-potential analysis, the purified samples were diluted in distilled water to a concentration of 200 μg mL^−1^ and briefly sonicated prior to the analysis. The results were expressed as mean ± standard deviation (SD) of three measurements.

#### Morphology

Transmission electron microscopy (TEM) images of hNPs were obtained on a Jeol JEM 2100Plus (Jeol, Tokyo, Japan) electron microscope, operating with an acceleration voltage of 200 kV and equipped with a 9 MP complementary metal oxide superconductor (CMOS) Gatan Rio9 digital camera (Gatan, Inc., Pleasanton, CA, USA). The samples were prepared by evaporating 5 μL of 1 mg mL^−1^ hetero-NPs onto carbon-coated copper grid (200 mesh) and allowing it to dry on the air. hNPs were positively stained with 2% uranyl acetate in phosphate saline buffer (PBS).

#### Cell culture

HeLa cells were cultured in Dulbecco's Modified Eagle's Medium (DMEM) high glucose supplemented with 2 mM l-glutamine, penicillin (50 U mL^−1^), streptomycin (50 mg mL^−1^) and 10% of Fetal Bovine Serum (FBS). Cells were cultured at 37 °C in humidified atmosphere containing 5% CO_2_ and sub-cultured prior to confluence using trypsin/EDTA. Cell media and supplements were purchased from Euroclone (Pero, MI, Italy).

#### Cell viability assay (MTS assay)

HeLa cells were seeded on a 96-multiwell dish at a density of 5 × 10^3^ cells per well and grown for 24 h in the appropriate medium. Cells were then incubated with hNP1–4 at equivalent concentrations of PTX ranging from 50 μM to 800 nM, obtained by serial 1 : 2 dilutions. As control, cells were incubated with self-assembly inducer 5 and with free PTX 6 at concentrations ranging from 5 μM to 312 nM, obtained once again by serial 1 : 2 dilutions. After 72 h of incubation, 20 μL of 3-(4,5-dimethylthiazol-2-yl)-5-(3-carboxymethoxyphenyl)-2-(4-sulfophenyl)-2*H*-tetrazolium (MTS) stock solution (Promega, Milano, Italy) were added to each well, and cells were incubated for additional 3 h at 37 °C. Afterwards, the absorbances at 490 nm were measured with EnSight™ Multimode Plate Reader (PerkinElmer, Waltham, MA, USA) and cell viability was calculated normalizing the detected Abs against the one recorded in the untreated sample. Results were expressed as mean ± standard deviation (*n* = 3). Each IC_50_ was calculated as the concentration that reduced cell viability by 50% after setting both a shared bottom and top constrains for all samples.

## Conflicts of interest

There are no conflicts to declare.

## Supplementary Material

RA-012-D2RA06306A-s001
