# Supplementary material for: Folic acid functionalization for targeting self-assembled paclitaxel-based nanoparticles
Source: RSC Adv. 2022 Dec 12;12(54):35484–93. doi: 10.1039/d2ra06306a (PMC9744106; doi:10.1039/d2ra06306a)

Electronic Supplementary Material (ESI) for RSC Advances.
This journal is © The Royal Society of Chemistry 2022

Supplementary Information

Folic Acid Functionalization For Targeting Self-Assembled Paclitaxel-Based Nanoparticles

Eleonora Colombo,[a,b] Davide Andrea Coppini,[a] Simone Maculan,[a] Pierfausto Seneci,[a] Benedetta Santini,[a] Filippo Testa,[c] Lucia Salvioni,[c] Giovanni Maria Vanacore,[d] Miriam Colombo, [c] and Daniele Passarella*[a]

[a.] Dipartimento di Chimica, Università degli Studi di Milano, Via Golgi 19, 20133 Milano, Italy. E-mail: daniele.passarella@unimi.it,

[b.] Ann Romney Center for Neurologic Diseases, Department of Neurology, Brigham and Women's Hospital and Harvard Medical School, Boston, MA 02115, USA

[c.] Dipartimento di Biotecnologie e Bioscienze - Università degli Studi di Milano Bicocca - Piazza della Scienza 2, 20126 Milano, Italy

[d.] Dipartimento di Scienza dei Materiali - Università degli Studi di Milano Bicocca - Via Roberto Cozzi 55, 20125, Milano, Italy

Sommario

^{1}H and ^{13}C Spectra 3

^{1}H NMR of 3 3

^{1}H NMR of 5 4

^{1}H and ^{13}C NMR of 1 5

^{1}H NMR of 8 6

^{1}H and ^{13}C NMR of 9 7

^{1}H and ^{13}C NMR of 10 8

^{1}H and ^{13}C NMR of 11 9

^{1}H and ^{13}C NMR of 12 10

^{1}H NMR of 21 11

^{1}H NMR of 22 11

^{1}H NMR of 18 12

^{1}H NMR of 17 12

^{1}H NMR of 16 13

^{1}H NMR of 15 13

^{1}H and ^{13}C NMR of 14 14

^{1}H and ^{13}C NMR of 2 15

^{1}H and ^{13}C Spectra

^{1}H NMR of compound 3

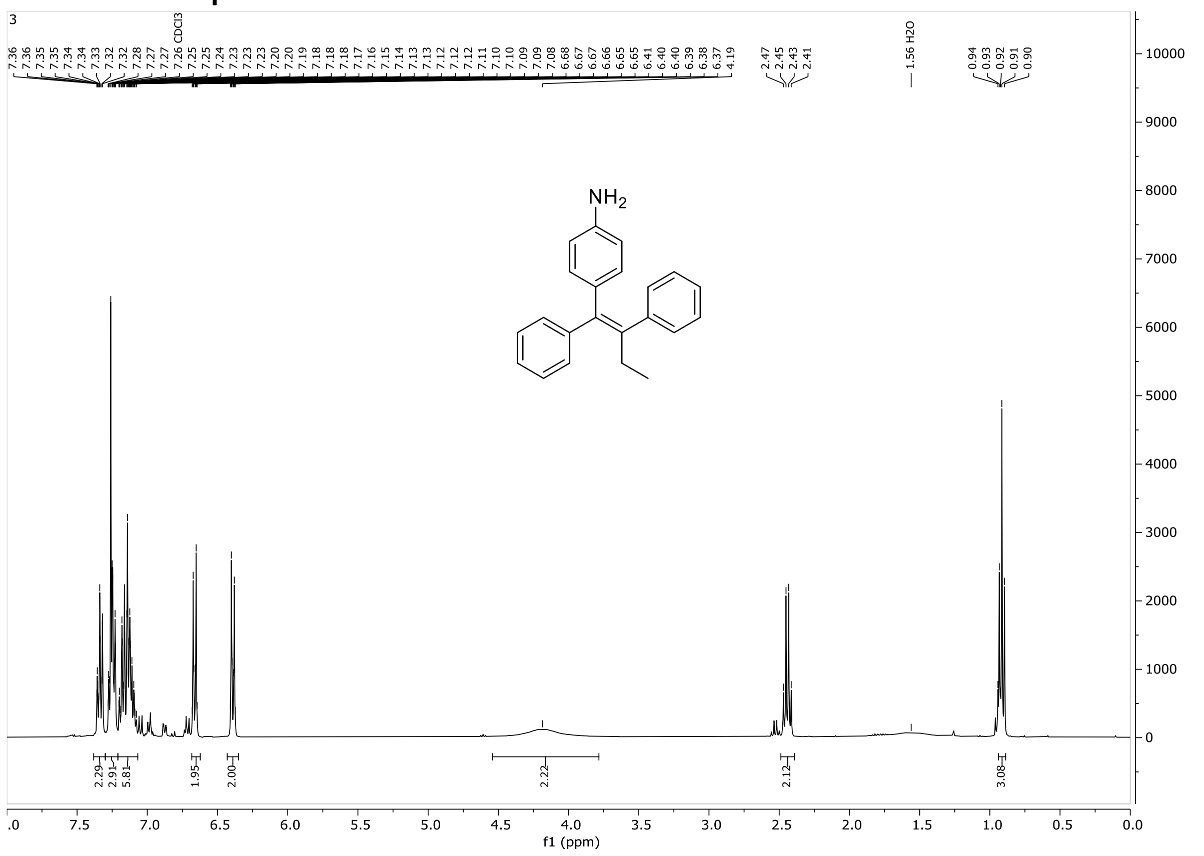

^{1}H NMR of 5

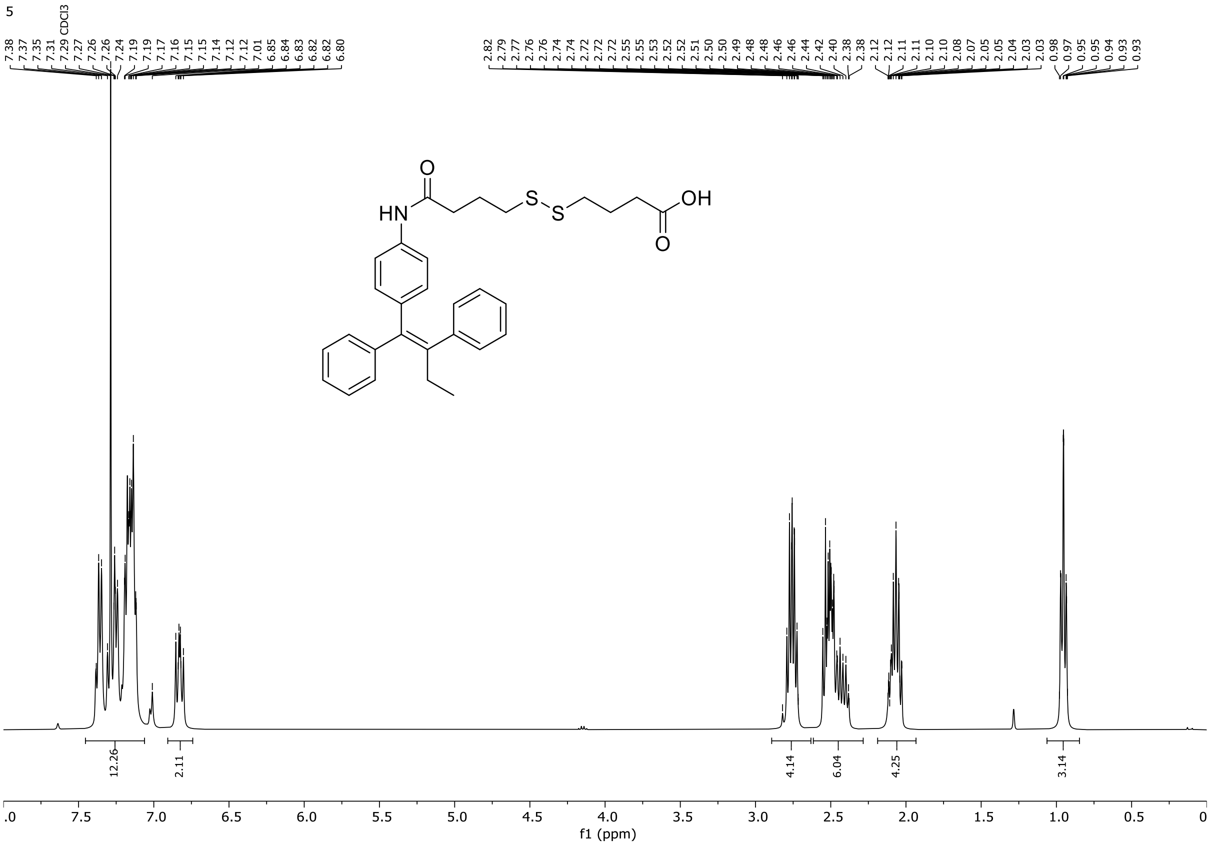
5
CDCl3
7.38 7.37 7.35 7.31 7.29 7.27 7.26 7.24 7.19 7.19 7.17 7.16 7.15 7.14 7.12 7.12 7.01 6.85 6.84 6.83 6.82 6.80
2.82 2.79 2.77 2.76 2.74 2.74 2.72 2.72 2.72 2.55 2.55 2.53 2.52 2.52 2.51 2.50 2.50 2.49 2.48 2.48 2.46 2.46 2.44 2.42 2.40 2.38 2.38 2.12 2.12 2.11 2.11 2.10 2.10 2.08 2.07 2.05 2.05 2.04 2.03 2.03 0.98 0.97 0.95 0.95 0.94 0.93 0.93
O
HN
S
S
OH
O
12.26
2.11
4.14
6.04
4.25
3.14
f1 (ppm)

^1H and ^{13}C-APT NMR of compound 1

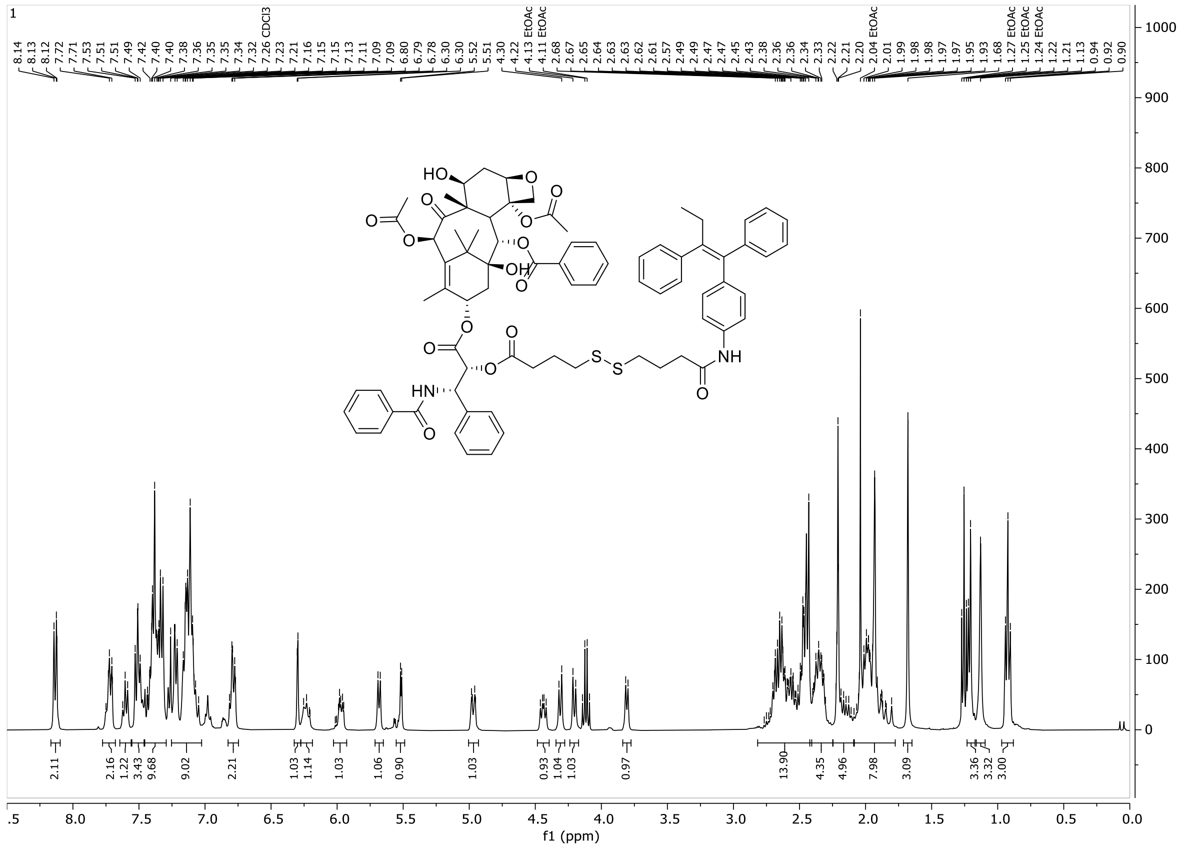

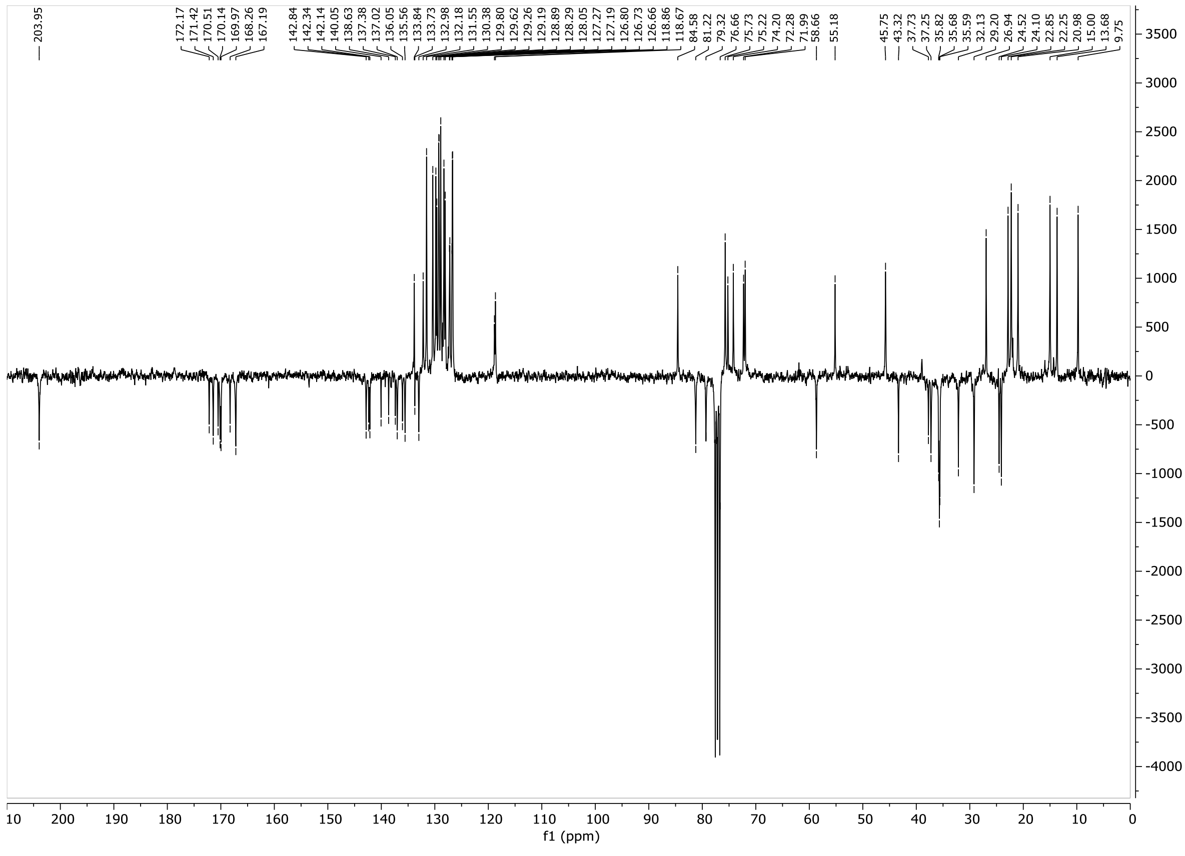

^{1}H NMR of 8

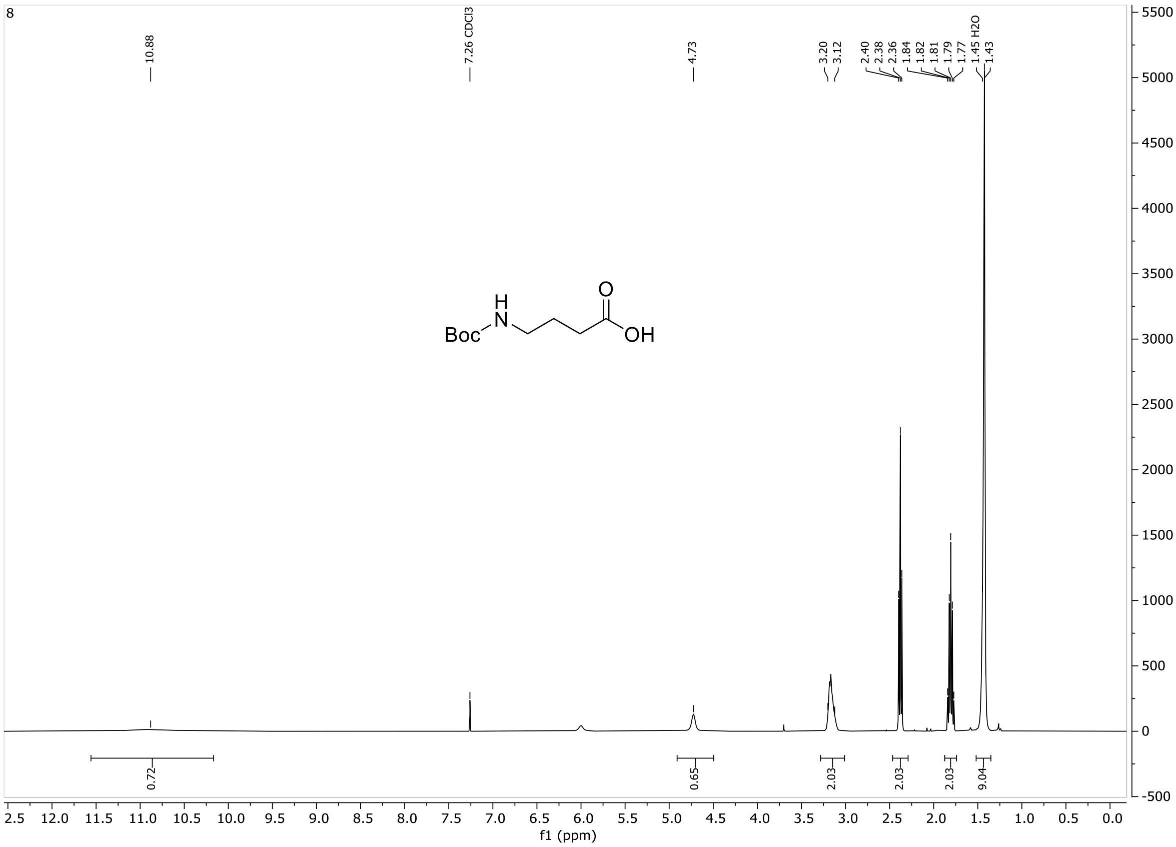

8
10.88
7.26 CDCl3
4.73
3.20
3.12
2.40
2.38
2.36
1.84
1.82
1.81
1.79
1.77
1.45 H2O
1.43
Boc
H
N
O
OH
0.72
0.65
2.03
2.03
2.03
9.04
f1 (ppm)

^{1}H and ^{13}C NMR of 9

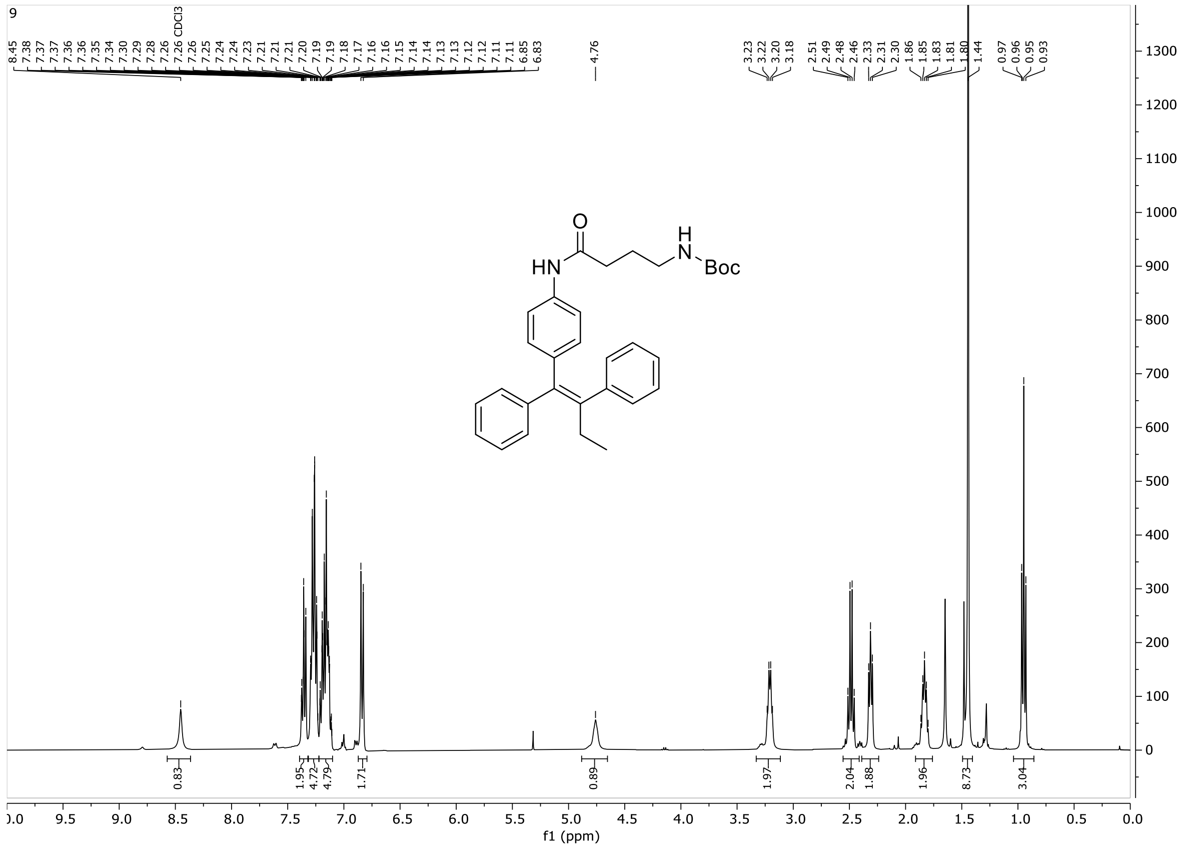

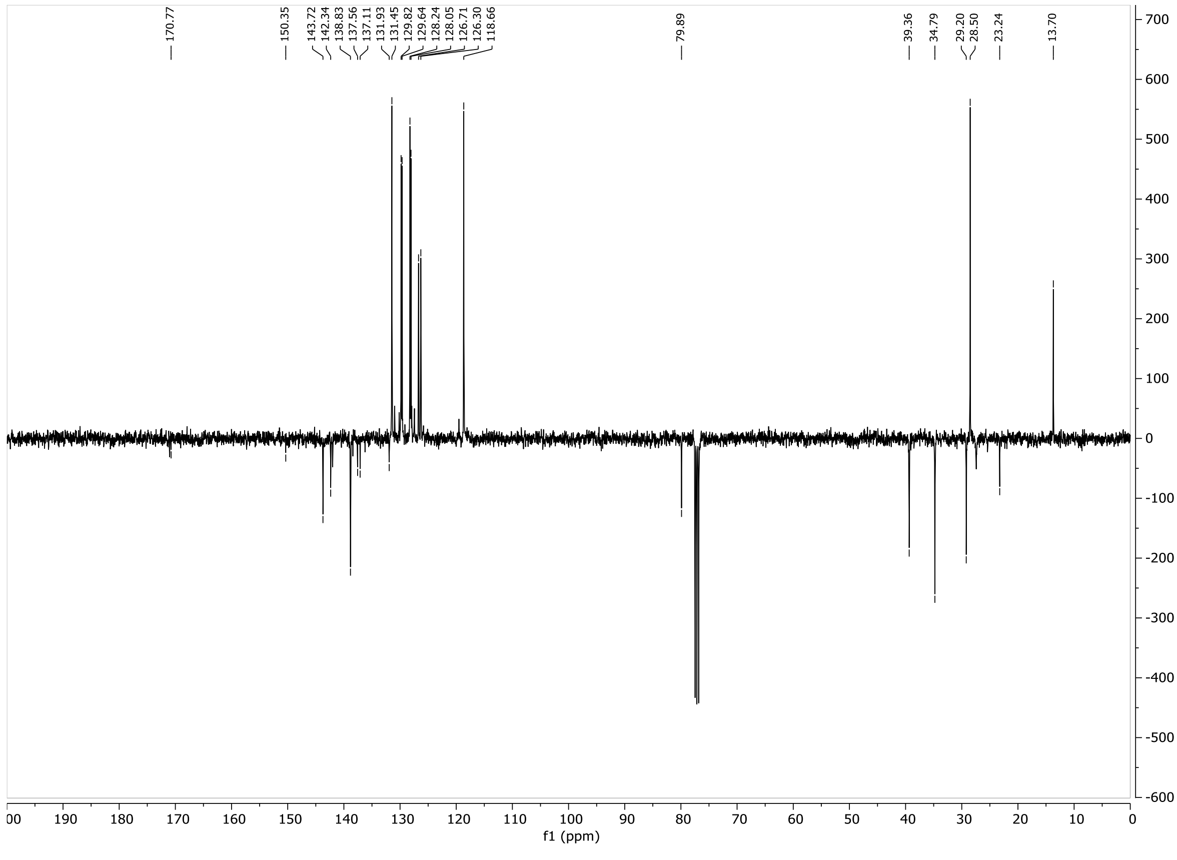

^{1}H and ^{13}C NMR of 10

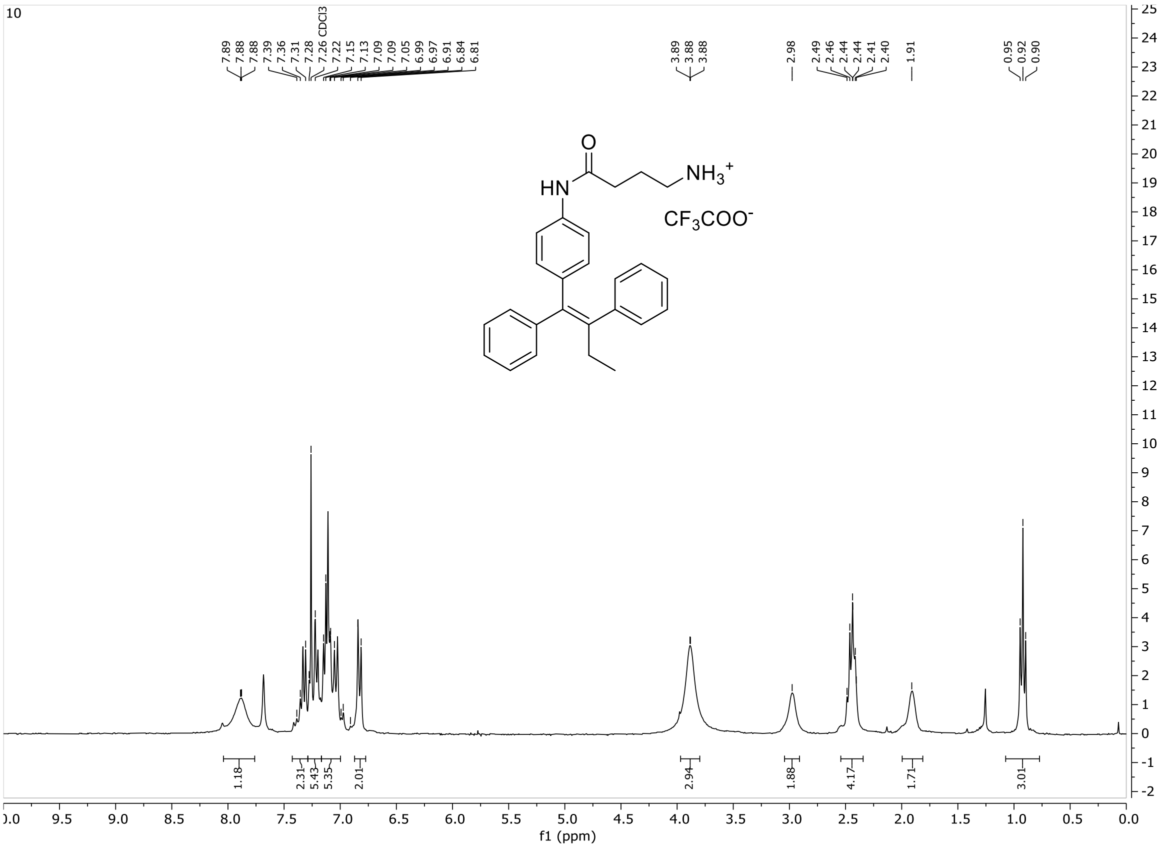

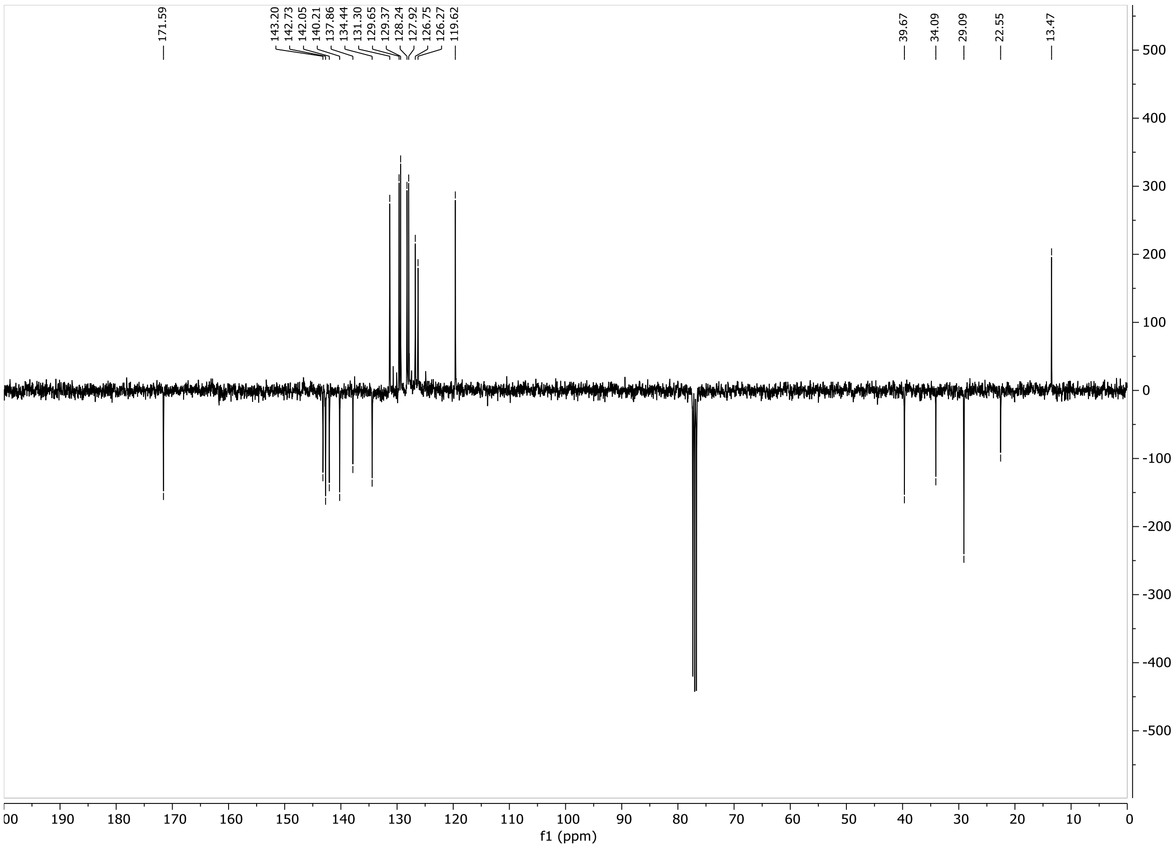

^{1}H and ^{13}C NMR of 11

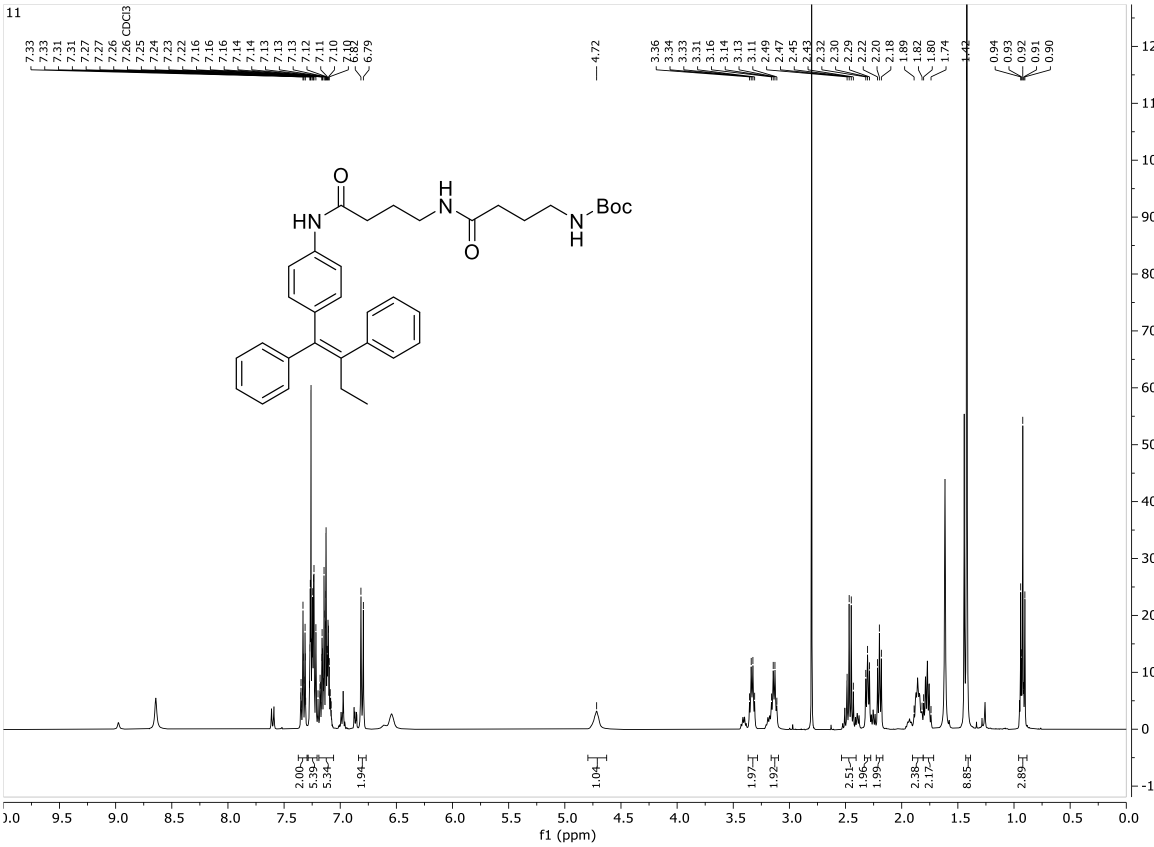

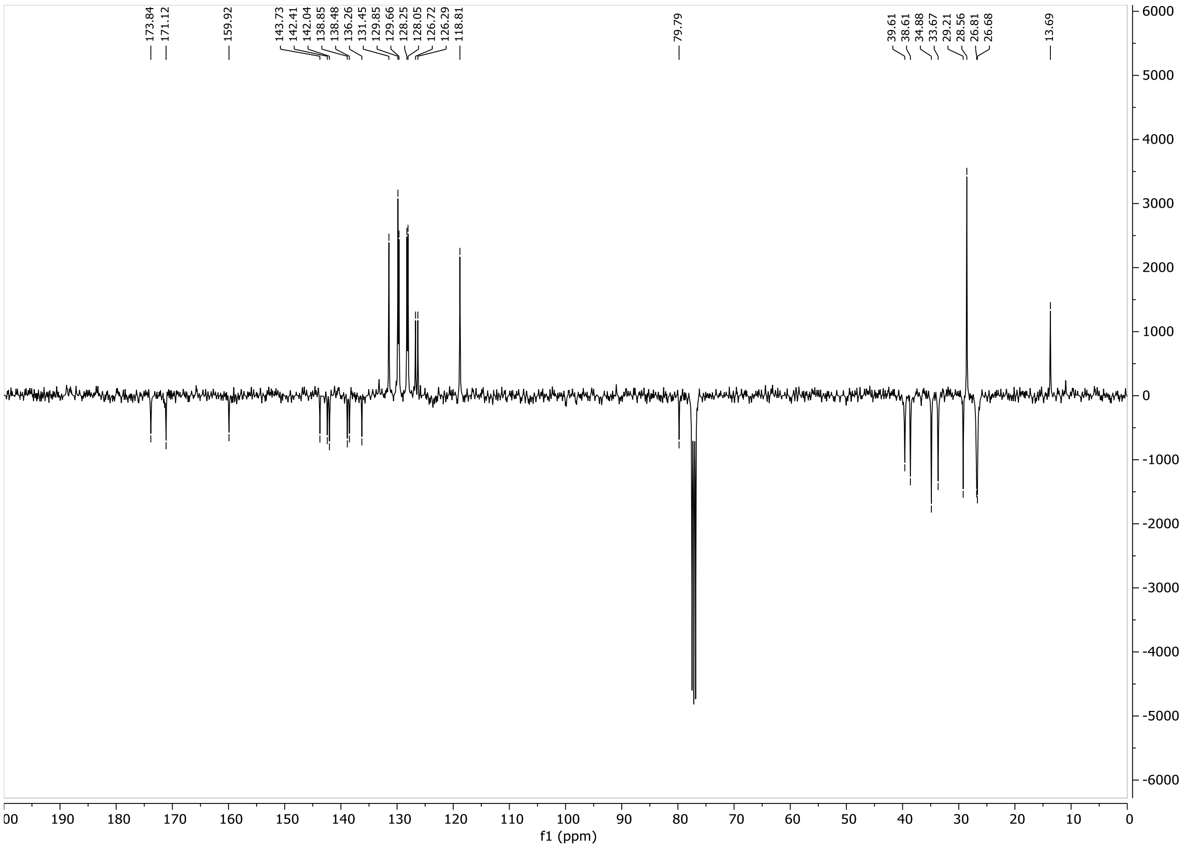

^{1}H and ^{13}C NMR of 12

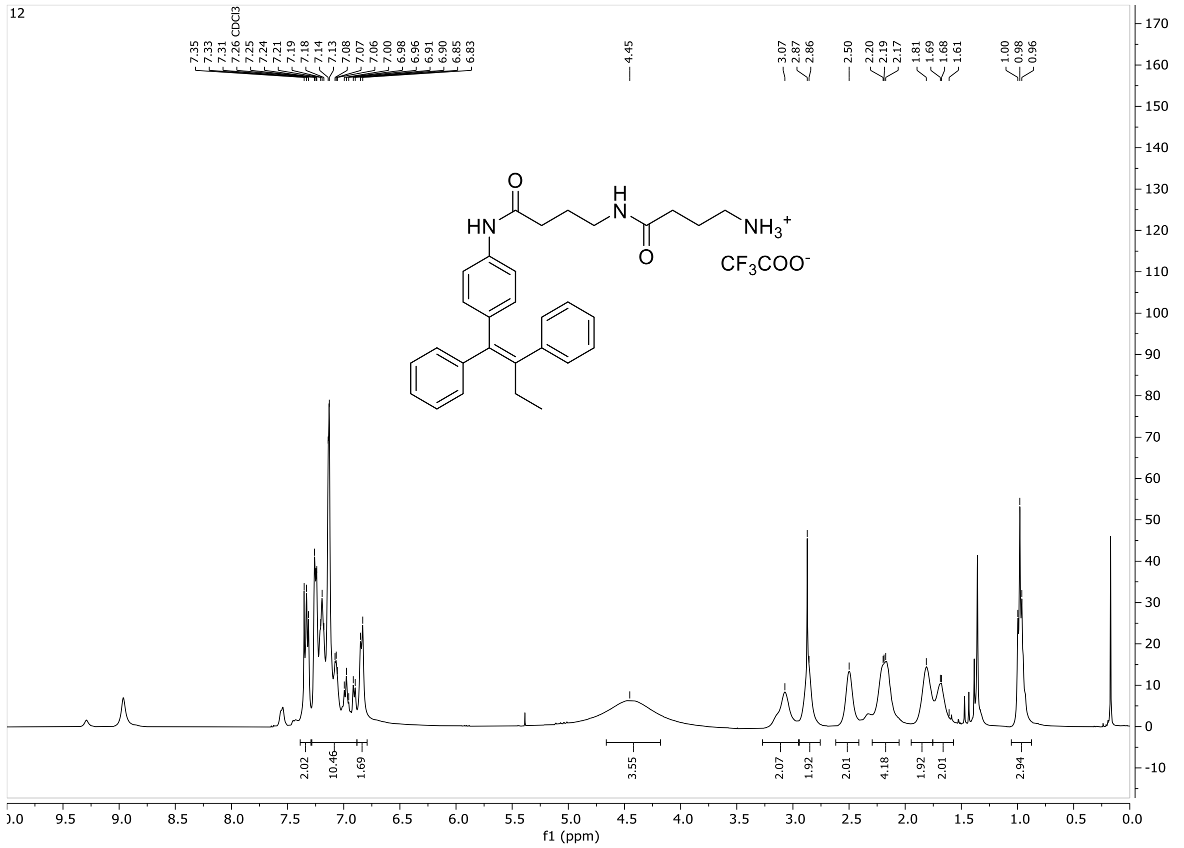

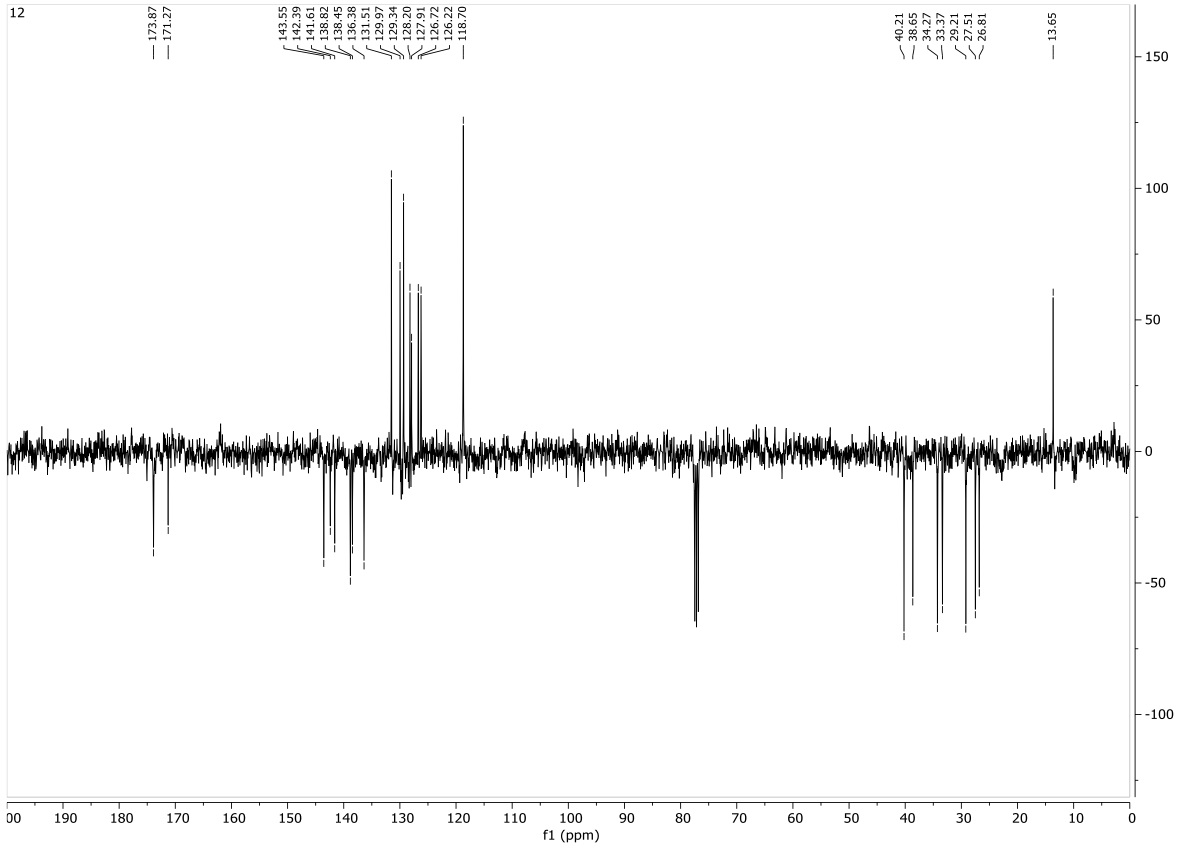

^{1}H NMR of 21

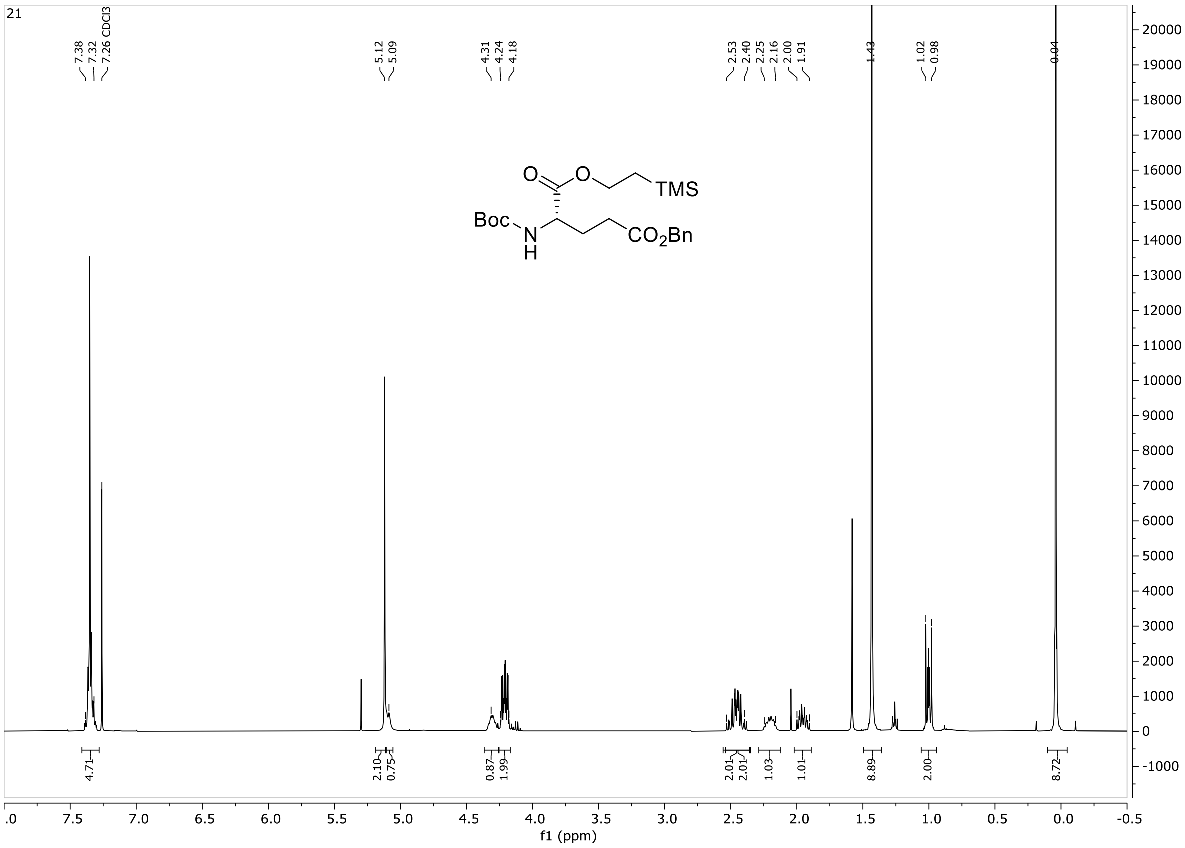

^{1}H NMR of 22

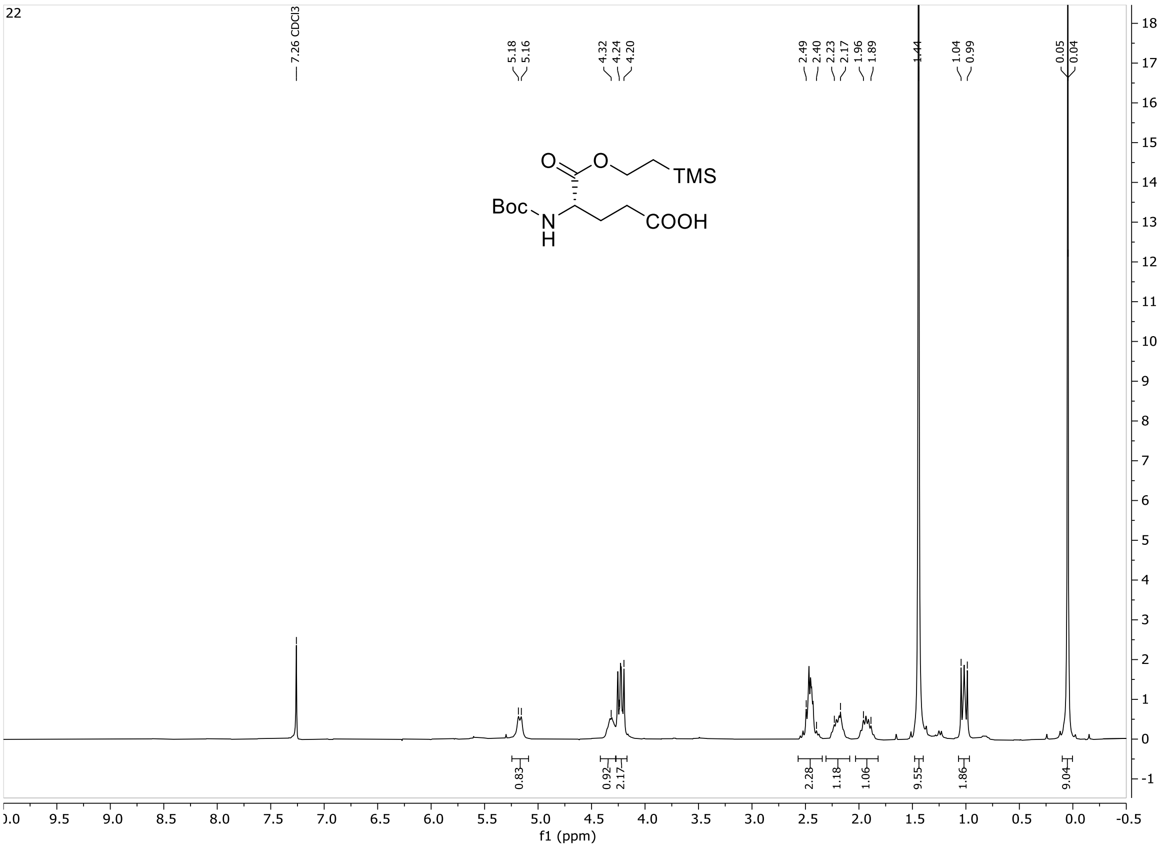

^{1}H NMR of 18

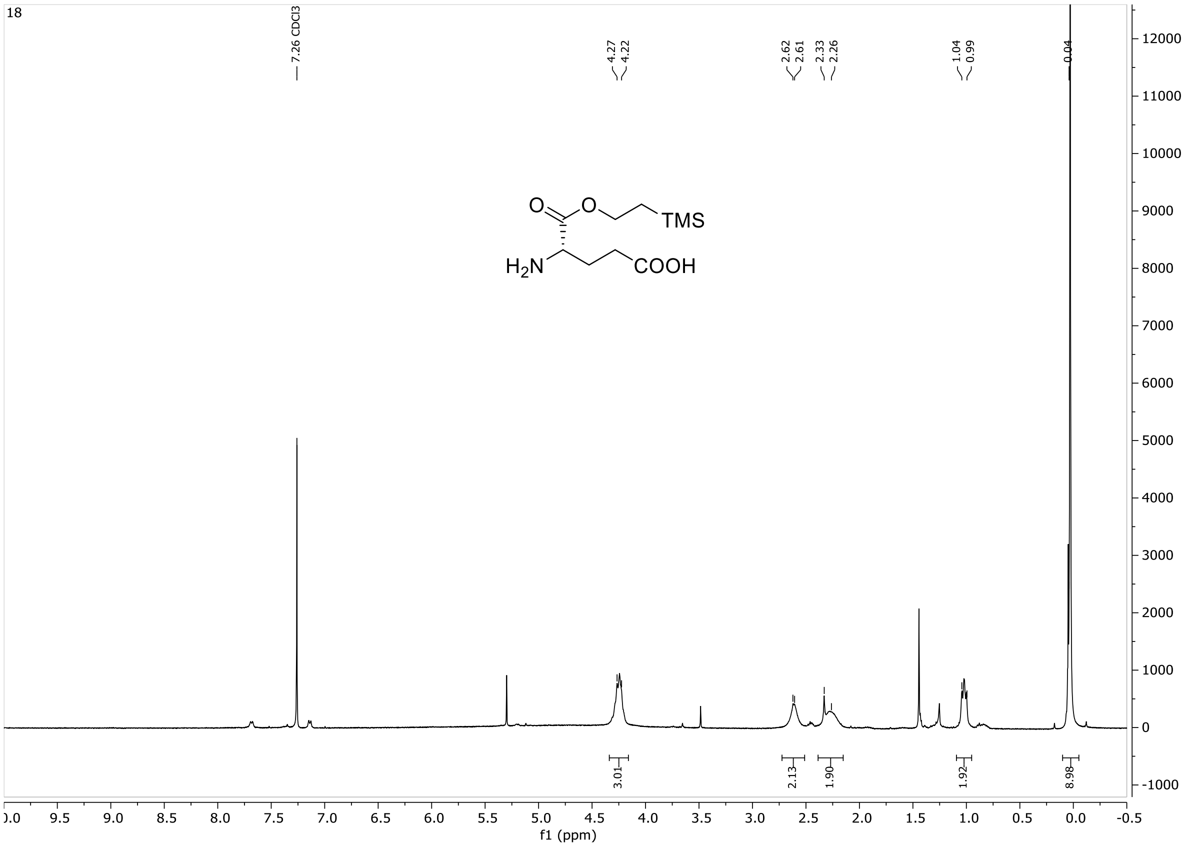

^{1}H NMR of 17

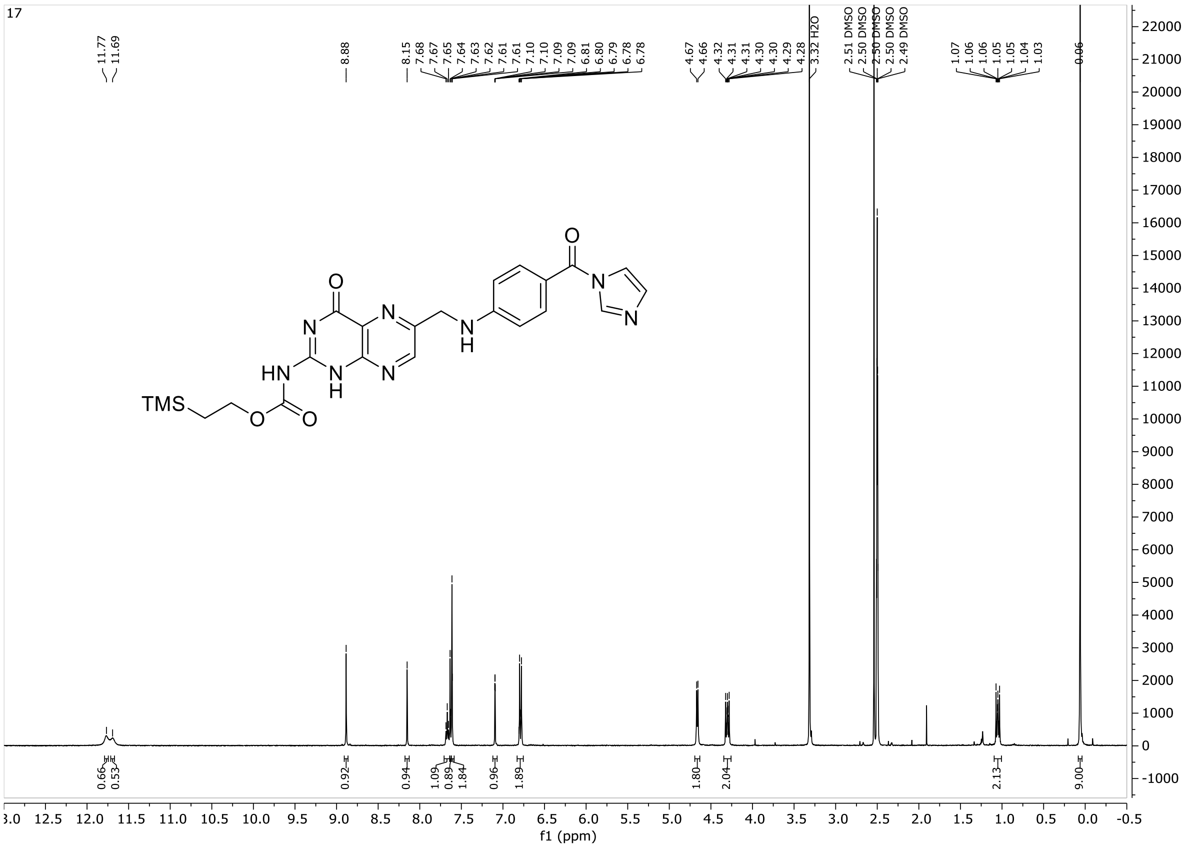

^{1}H NMR of 16

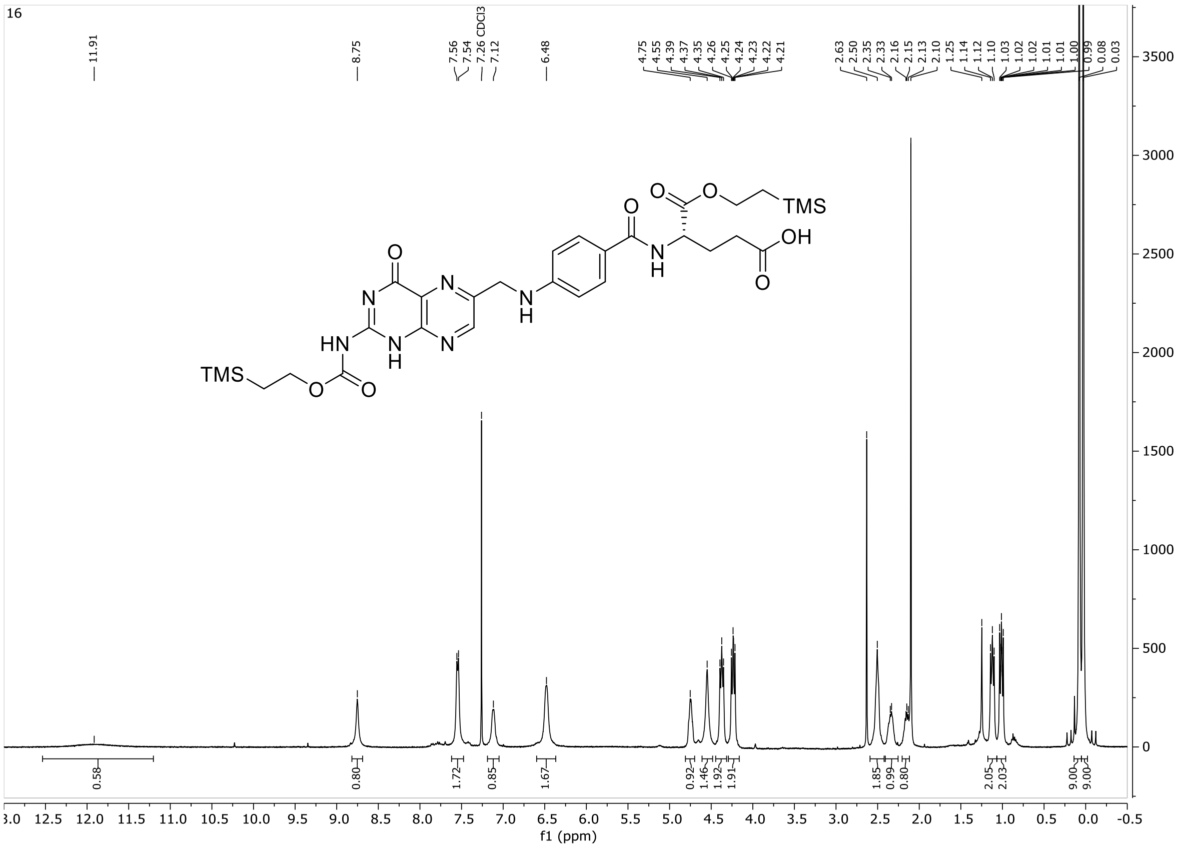

^{1}H NMR of 15

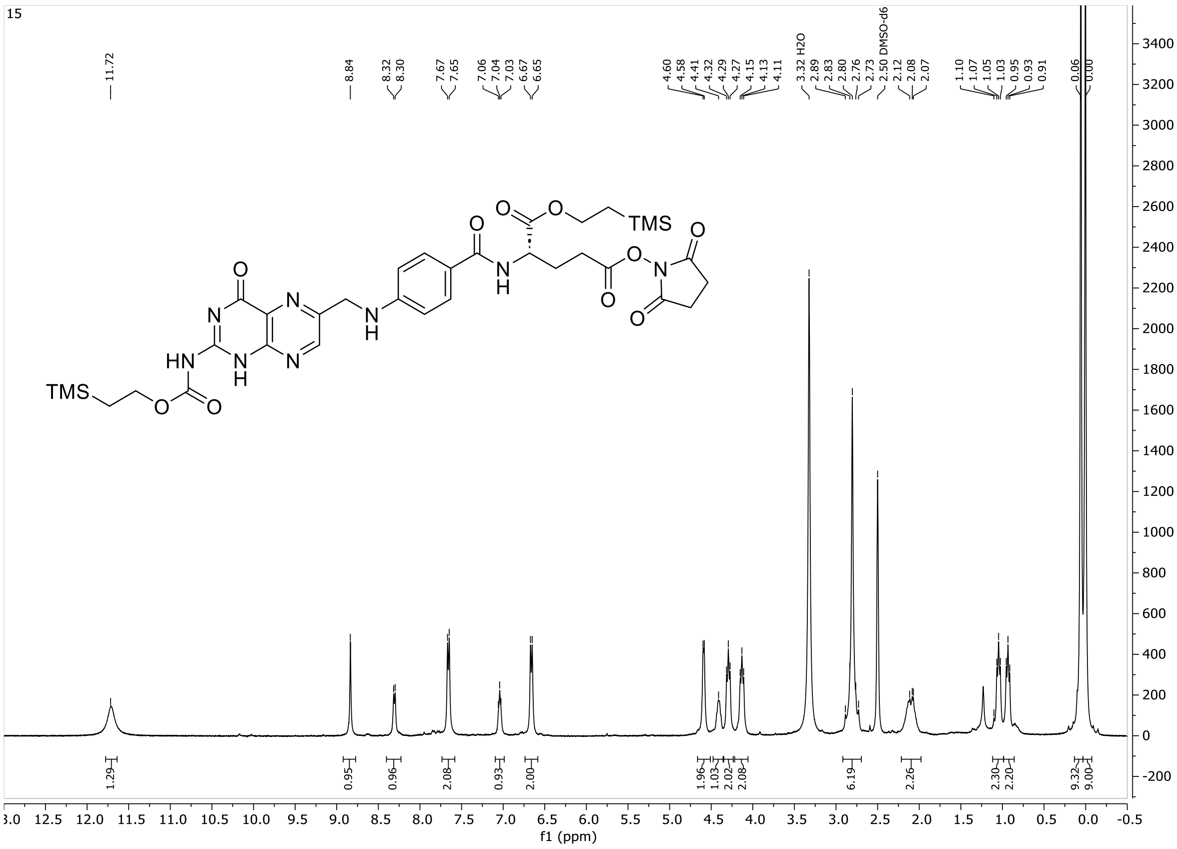

^{1}H and ^{13}C NMR of 14

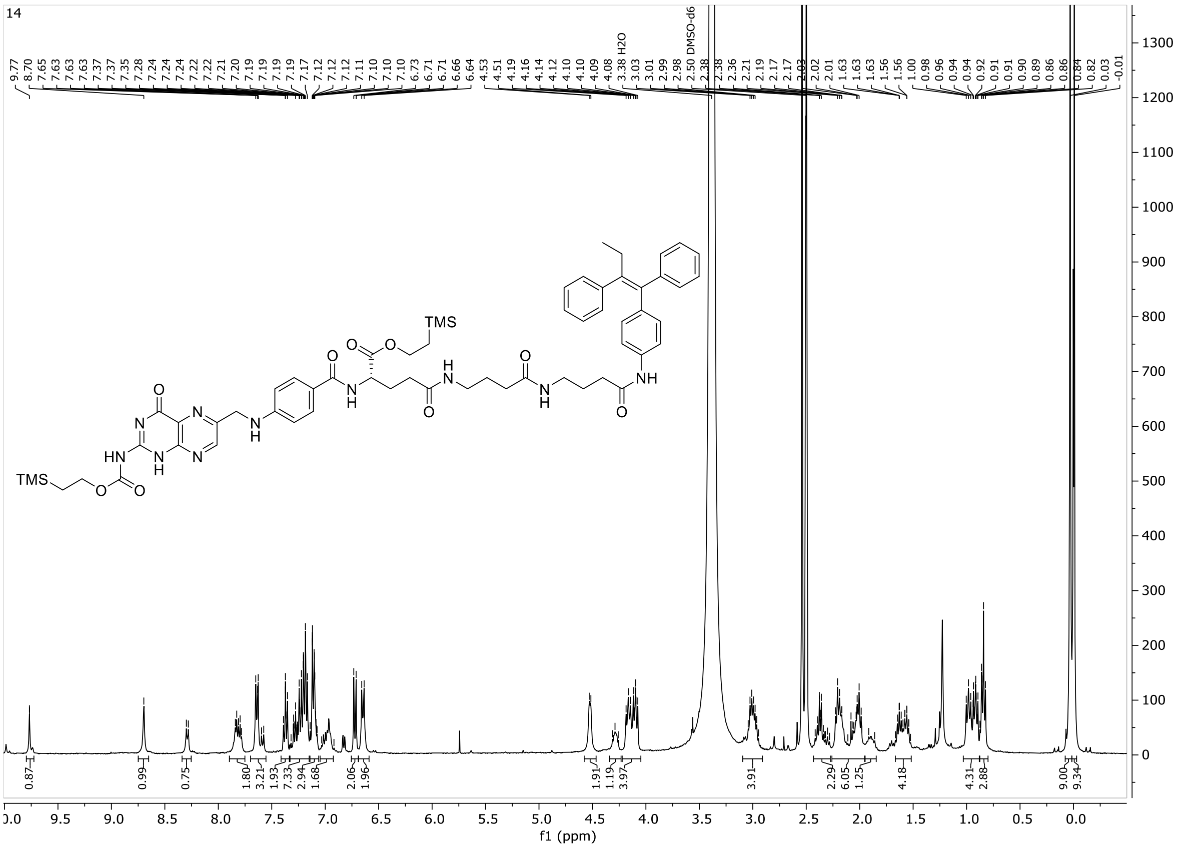

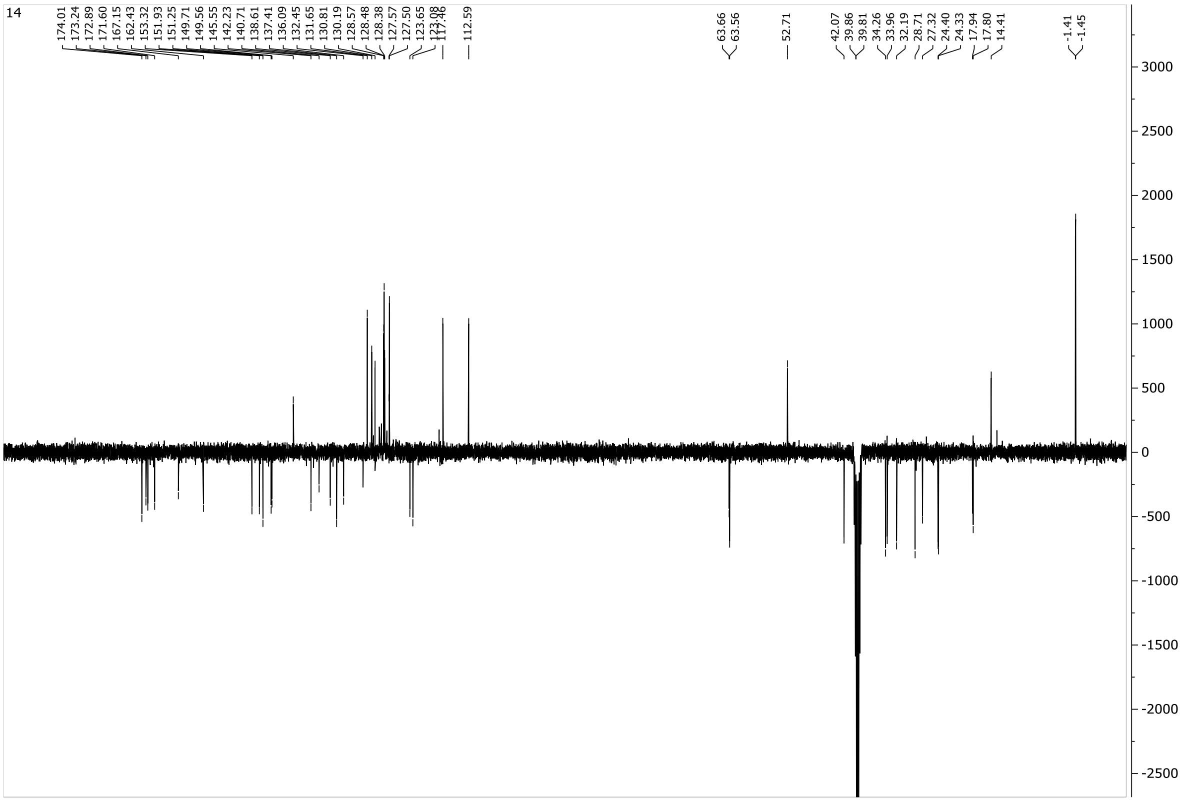

^{1}H and ^{13}C NMR of 2

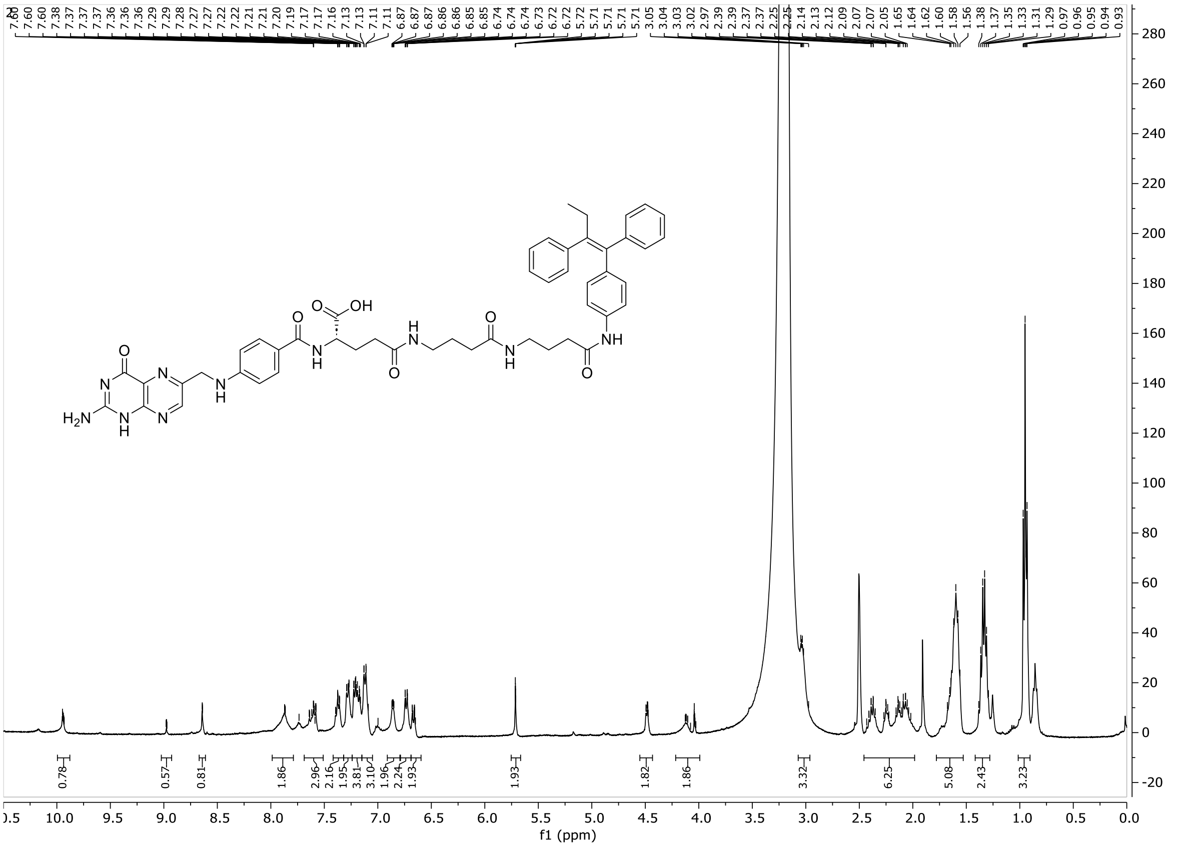

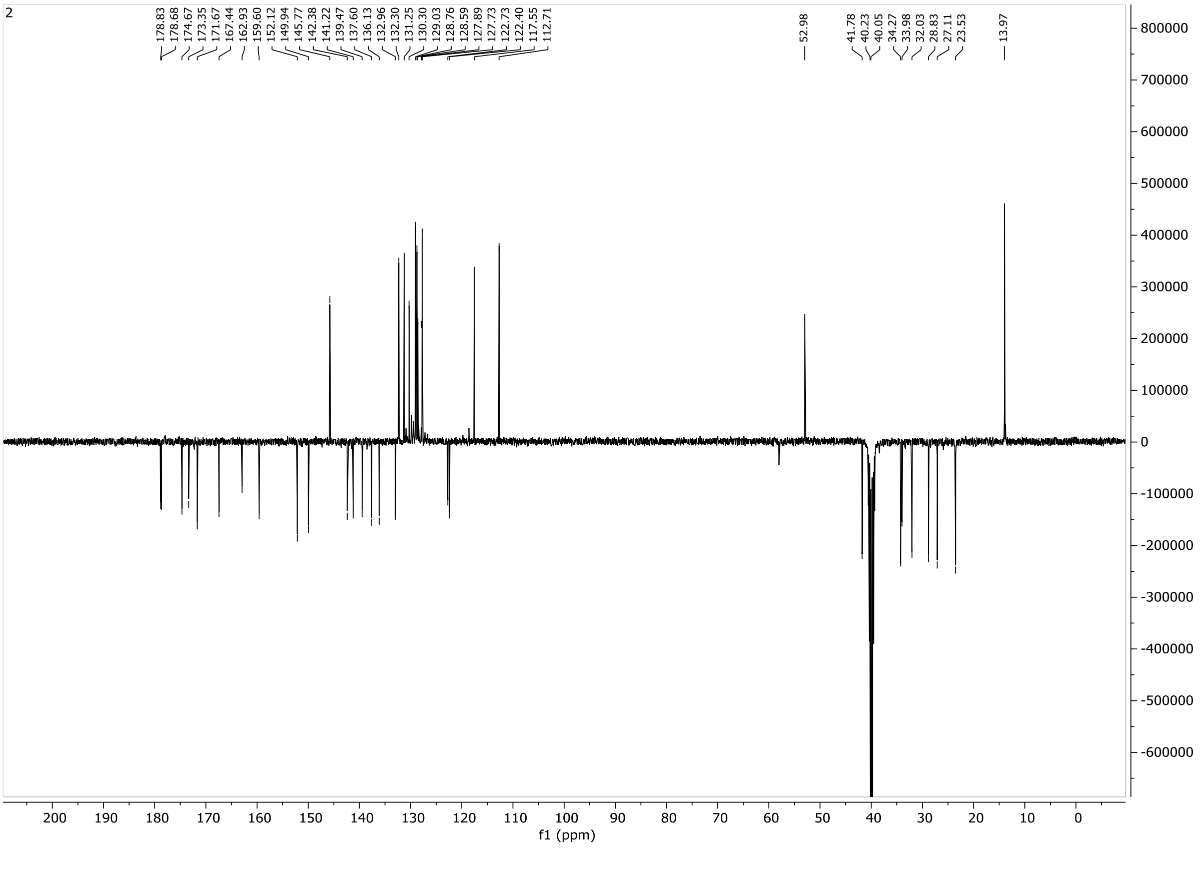

Supplement: RA-012-D2RA06306A-s001 [file RA-012-D2RA06306A-s001.pdf]
